# MiR146a-loaded engineered exosomes released from silk fibroin patch promote diabetic wound healing by targeting IRAK1

**DOI:** 10.1038/s41392-022-01263-w

**Published:** 2023-02-13

**Authors:** Qiankun Li, Wenzhi Hu, Qilin Huang, Jie Yang, Bingmin Li, Kui Ma, Qian Wei, Yaxi Wang, Jianlong Su, Mengli Sun, Shengnan Cui, Rungong Yang, Haihong Li, Xiaobing Fu, Cuiping Zhang

**Affiliations:** 1 Department of Tissue Repair and Regeneration, The First Medical Center, Chinese PLA General Hospital, Beijing, 100853 China; 2 Research Center for Tissue Repair and Regeneration, Medical Innovation Research Department and the Fourth Medical Center, Chinese PLA General Hospital, Beijing, 100048 China; 3 Dermatology Department, The Fourth Medical Center, Chinese PLA General Hospital, Beijing, 100048 China; 4 Research Unit of Trauma Care, Tissue Repair and Regeneration, Chinese Academy of Medical Sciences, 2019RU051, Beijing, 100048 China; 5 PLA Key Laboratory of Tissue Repair and Regenerative Medicine and Beijing Key Research Laboratory of Skin Injury, Repair and Regeneration, Beijing, 100048 China; 6 School of Medicine, NanKai University, Tianjing, 300071 China; 7 Department of Wound Repair and Dermatologic Surgery, Taihe Hospital, Hubei University of Medicine, Shiyan, 442000 China

**Keywords:** Nucleic-acid therapeutics, Cell delivery, Biomaterials

## Abstract

Unhealable diabetic wounds need to be addressed with the help of newer, more efficacious strategies. Exosomes combined with biomaterials for sustained delivery of therapeutic agents are expected to bring new hope for chronic wound treatment. Here, the engineered exosomes modified for efficiently loading miR146a and attaching to silk fibroin patch (SFP) were demonstrated to promote diabetic wound healing. Silk fibroin binding peptide (SFBP) was screened through phage display, and SFBP-Gluc-MS2 (SGM) and pac-miR146a-pac fusion protein were constructed. The designed exosomes (SGM-Exos, miR146a-Exos, and SGM-miR146a-Exos) were isolated from the engineered placental mesenchymal stem cells (PMSCs) transduced with SGM or/and pac-miR146a-pac protein. Gluc signals indicated SGM-Exo@SFP markedly increased the binding rate and the stability of SGM-Exo. Moreover, the loading efficiency of miR146a in SGM-miR146a-Exos was ten-fold higher than that in miR146a-Exos. Superior to untreated, SGM-miR146a-Exo-only treated, and SFP-only treated groups, SGM-miR146a-Exo@SFP drived wound healing associated with less inflammation, collagen deposition, and neovascularization. The transcriptomics analysis suggested anti-inflammatory and regenerative effects with SGM-miR146a-Exo@SFP treatment. Here, we show efficient exosome@biomaterial-based miRNA delivery systems for regenerative medicine and tissue engineering.

## Introduction

Extracellular vesicles (EVs) or exosomes, membrane-enclosed and secreted by numerous cell types,^1^ have become an increasingly attractive field of research in recent years, with applications in treatment for a wide range of diseases. Initially, EVs were thought as “cellular dust” and not having any value, primarily to function in the maintenance of cellular homeostasis by the waste removal from cells. In 1996, Raposo et al.^2^ described the first evidence of EVs playing important biological functions. In 2005, Valadi et al.^3^ demonstrated that EVs can transfer functional mRNA molecules to recipient cells and thereby mediate cell–cell communication. Subsequently, a wealth of evidence indicated that EVs played significant physiological roles, in addition to their pathological roles, through the delivery of bioactive molecules, such as RNA, proteins, lipids, and others.^4^ With an increasing understanding of EV characteristics, the potential utility of EVs in tissue engineering and regenerative medicine has been explored through direct application or via incorporation into biomaterial scaffolds.

In vivo, EVs have a high potential for absorption by neighboring cells and therefore a very short half-life, instability, and low long-term retention after transplantation. Incorporation of EVs into biomaterials may be a powerful tool for enhancing EV stability and realizing a controlled sustained release within the body.^5^ This premise is supported by several studies on incorporating EVs into biomaterial constructs for delivery.^6^ But it’s worth noting that the ability of biomaterials hosting EVs is different, which maybe depend on the binding efficiency of EVs to biomaterials. Scientists have found evidence that EVs can combine with extracellular matrix (ECM) components^7^ in a similar manner to the sequestration of growth factors in ECM. Adhesion receptors presented on the exosomal membrane are likely responsible for the interactions between EVs and ECM, implying that integrating instructive biomolecular signals into exosomal membrane targeting biomaterials can enhance the binding rate of EVs to biomaterials.

MicroRNAs (miRNAs), as part of exosomal cargo, can bind to their target mRNAs negatively regulating mRNA expression posttranscriptionally.^8^ Due to the instability and susceptibility to degradation, miRNAs are difficult to be transferred into target cells, warranting the development of an effective miRNA delivery system.^9,10^ EVs have been recognized as promising vehicles for biomolecules delivery owing to their ability to protect them from serum proteases and immune responses. For delivery of the nucleic acids (i.e., siRNAs, miRNAs etc.), different methods have been developed to package them into EVs, such as electroporation, sonication, chemical transfection, etc.^11^ However, certain limitations associated with these methods may include compromised integrity and stability of the engineered EVs as well as decreased biological activity of the cargo.^12,13^ Biological methods employed for miRNA-loaded exosomes from genetically modified host cells have gained significant attention during the past few years,^14^ with lacking undesired side effects and maintaining the structural integrity of the exosomal membrane. But how to encapsulate miRNAs efficiently in engineered exosomes by engineering parent cells is a big challenge for better therapeutic effects.

With the advent of the global aging society, the number of patients with diabetes is increasing, and refractory wounds such as diabetic foot and diabetic ulcer are getting more and more attention as the complications of patients with diabetes.^15^ The normal wound healing process involves the phases of hemostasis, inflammation, proliferation, and remodeling. A chronic wound usually results owning to halt in the phase of inflammation followed by impaired angiogenesis and delayed re-epithelialization.^16,17^ The basic cause of it is persistent/chronic bacterial infection and biofilm formation. Therefore, preventing infection and controlling inflammation is critical to chronic wound treatment. Among wound dressing biomaterials, silk fibroin (SF) has attracted significant attention for wound healing because of antimicrobial properties to prevent pathogen invasion and proliferation, thereby reducing the risk of wound infection.^18^ Exosomes derived from various stem cells have been indicated to promote skin repair and regeneration,^19^ but seldom of them have antimicrobial function, which suggests incorporating EVs into SF scaffold for the treatment of chronic wounds. However, most of native exosomes usually contain less anti-inflammation active components, therefore having limited therapeutic effects on infective wounds. One possible solution to this limitation is engineered exosomes, which could be used to encapsulate anti-inflammatory regulator such as miRNA-146a (miR146a)^20,21^ via genetic engineering.

Here, we report a novel and efficient miRNA delivery system based on engineered exosomes and SF scaffold to promote diabetic wound healing. Silk fibroin binding peptide (SFBP)-Gluc-MS2 (SGM) and pac-miR146a-pac fusion proteins were constructed and transduced into human placenta-derived MSCs (PMSCs). Subsequently, the SGM protein could be integrated into plasma membrane or endosomal membrane of PMSCs. When exosomes are shed from the membranes, miR146a will be encapsulated efficiently in exosomes by the specific binding of phage MS2 capsid protein and pac site.^22^ Furthermore, during the process of exosome biogenesis, SFBP targeting SF was expressed into exosomal membrane, therefore enhancing the binding rate of SGM-miR146a-Exos to SFP. The system developed here could improve the efficiency of engineered exosomes carrying target miRNA through MS2 in order to play a regulatory role more effectively. Besides, silk fibroin affinity exosomes bound to SF could be better preserved and maintain lasting therapeutic effects. Compared with untreated, SGM-miR146a-Exo-only treated, and SFP-only treated groups, SGM-miR146a-Exo@SFP achieved a better therapeutic effect on diabetic wound healing.

## Results

### Characterization of the engineered exosomes

**Fig. 1:**
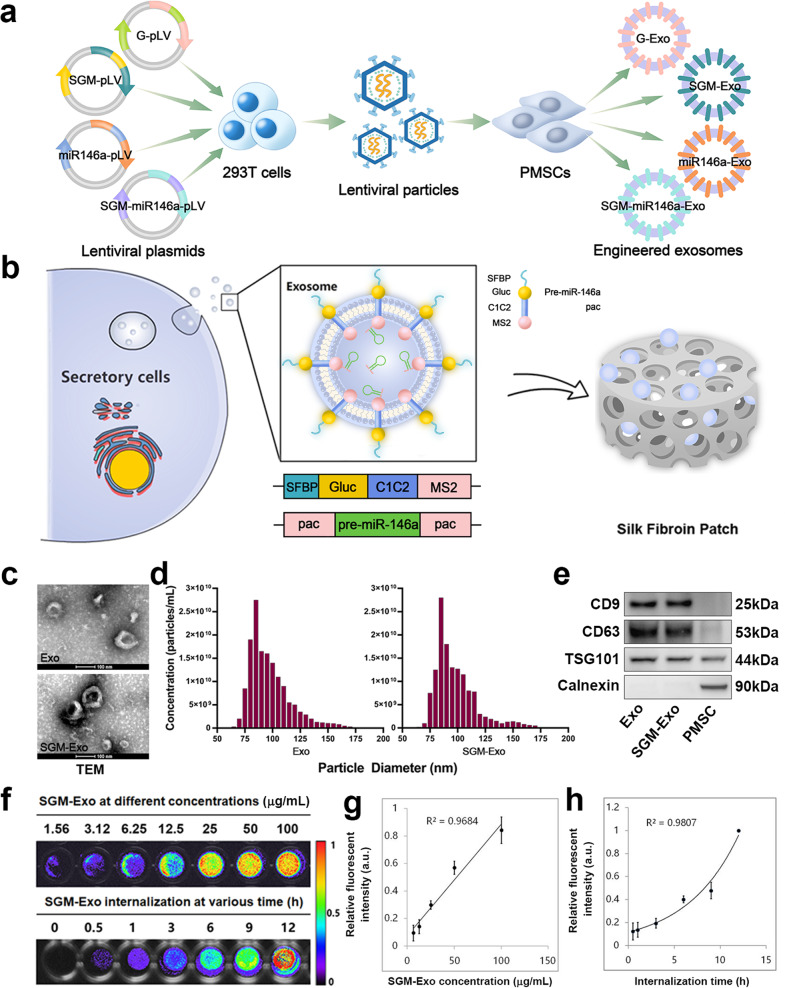
Generation and characterization of engineered exosomes. **a** Schematic diagram representing the generation of designed engineered exosomes including G-Exo, SGM-Exo, miR146a-Exo, and SGM-miR146a-Exo. **b** Schematic of donor cells (left). Schematic of DNA constructs used for the production of SGM-miR146a-Exo (middle). Schematic of silk fibroin patch (right). **c** Representative transmission electron microscope images for Exos and SGM-Exos (scale bar: 100 nm). **d** Particle size distributions of Exos and SGM-Exos measured by dynamic light scattering (*n* = 4/group). **e** Western blot analysis of exosome markers. **f** Representative imaging of bioluminescence signals of SGM-Exos at different concentrations (upper). Representative imaging of bioluminescence signals of SGM-Exos internalized by HaCaT cells (lower). **g** Graph showing quantification of SGM-Exos at different concentrations (*n* = 4/group, *R*^2^ = 0.9684). **h** Graph showing quantification of SGM-Exos internalized by HaCaT cells (*n* = 4/group, *R*^2^ = 0.9807)

SFBP was selected through screening the 12-phage peptide library and validated by ELISA (Supplementary Fig. S1, Tables S1–S9). Next, we inserted SFBP between SP and C1C2 domains of exosomal membrane protein Lactadherin, and fused a bioluminescence report system Gaussia luciferase (Gluc)^23^ following SFBP to monitor exosomes in real-time. MS2 domain was fused to the C terminal of Lactadherin to cargo designed miRNA which have two pac sites at flanks. The coding sequences of fusion proteins were showed in Supplementary Tables S10–S15 and the construction of the functional fusion protein SGM was displayed in Supplementary Table S16. The constructed lentiviral plasmids (pLV) were Gluc-pLV (G-pLV), SFBP-Gluc-MS2-pLV (SGM-pLV), pac-miR146a-pac-pLV (miR146a-pLV) and SGM-miR146a-pLV respectively. The sequences of those lentiviral transfer plasmids were showed in Supplementary Fig. S3a. The lentiviral (Lv) particles were obtained from 293 T cells transfected with recombinant lentiviral plasmids. The obtained lentiviral particles were used to infect PMSCs (Supplementary Fig. S3b) and the stable transfected cell lines were established after screening by puromycin (Supplementary Fig. S3c). Subsequently, the designed engineered exosomes (SGM-Exo, miR146a-Exo, and SGM-miR146a-Exo) were isolated from supernatant of their parent PMSCs infected with SGM or/and pac-miR146a-pac Lv. The Gluc-labeled exosomes (G-Exo) were used as the control exosomes in luminescence imaging analysis (Fig. 1a). Then the engineered exosomes were incorporated into the SF scaffold for further treatment (Fig. 1b). Through TEM, DLS and WB analysis, we found that the characteristics of SGM-Exos were similar to that of Exos. As shown in Fig. 1c and d, both Exos and SGM-Exos displayed a typical cup or round shape appearance with a size of approximately 85 nm. Exosomal markers CD9, CD63, and TST101 were highly expressed in Exos and SGM-Exos detected by western blot. ER marker Calnexin, as a monitor of cellular contamination during exosome isolation, was only expressed in PMSCs while rarely detectable in Exos and SGM-Exos (Fig. 1e). Besides, the bioluminescence imaging (BLI) analysis (Fig. 1f) showed that the bioluminescence signals of SGM-Exo increased gradually with a dose-dependent manner (Fig. 1g) and SGM-Exos were steadily internalized over time by HaCaT cells with a concentration of 1 μg/mL (Fig. 1h).

### Preparation and characterization of SFP served as exosome-laden scaffolds

**Fig. 2:**
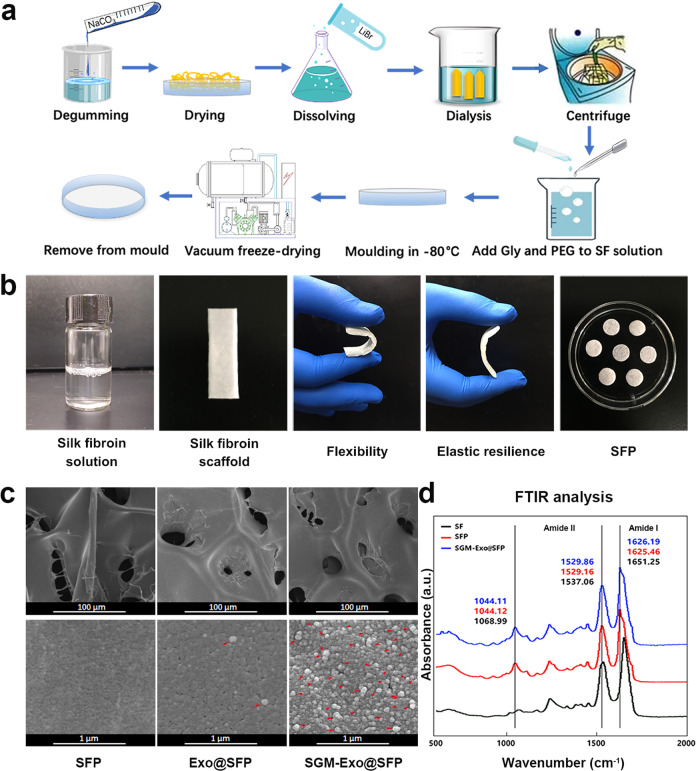
The preparation and characterization of the silk fibroin patch (SFP) served as exosome-laden scaffolds. **a** Schematic illustration of the preparation procedure for SF scaffolds. **b** Physical pictures of silk fibroin solution, SF scaffold, and SFP. **c** The structure characterizations of SFP, Exo@SFP, and SGM-Exo@SFP detected by scanning electron microscope (upper scale bars: 100 μm, lower scale bars: 1 μm). The red arrows indicated the exosomes loaded on the SFP. **d** FTIR spectra analysis of SF, SFP, and SGM-Exo@SFP (*n* = 4/group)

The preparation process to obtain SFP was shown schematically in Fig. 2a. As shown in Fig. 2b, the SFP was a light, soft and water-absorbing material with sufficient mechanical strength to pull and bend. The SFP was prepared according to the size of the wound. The favorable biological safety of SFP were evaluated to ensure security of application by the biocompatibility and cytotoxicity tests in vitro and in vivo (Supplementary Fig. S2). SFP had no influence on cell viability detected by CCK8 test. The blood biochemistry indicating hepatorenal function in SFP group were in the normal reference range. The histological sections of liver and kidney tissue showed no obvious abnormalities in SFP and control groups. To observe the structure characterizations of silk fibroin patch-exosome, Exos and SGM-Exos were respectively loaded on SFP and washed with deionized water. The SFP, Exo@SFP, and SGM-Exo@SFP were prepared for scanning electron microscope (SEM) detection. The images showed SFP had a loose texture with irregular or ribbon-like pores of different size being approximately 10–50 μm. Compared with the natural exosomes without the capacity of SF-affinity, the SF-affinity exosomes, SGM-Exos, distributed more densely on SFP (Fig. 2c). Next, the structures of SF, SFP, and SGM-Exo@SFP were analyzed by FTIR. SF had amide I and II peaks at 1651.25 and 1537.06 cm^−1^ representing random coil and α-helix structures respectively. However, the absorbance of SFP shifted to lower wavenumbers (1625.46 and 1529.16 cm^−1^) which represent the characteristics of protein β-sheet structure.^24,25^ Furthermore, we observed a slightly spectral shift to high wavelengths in SGM-Exo@SFP group, implying the engineered exosomes were successfully loaded onto the patch (Fig. 2d).

### Evaluation of binding rate, stability, and sustained release of SGM-Exos in SFP

**Fig. 3:**
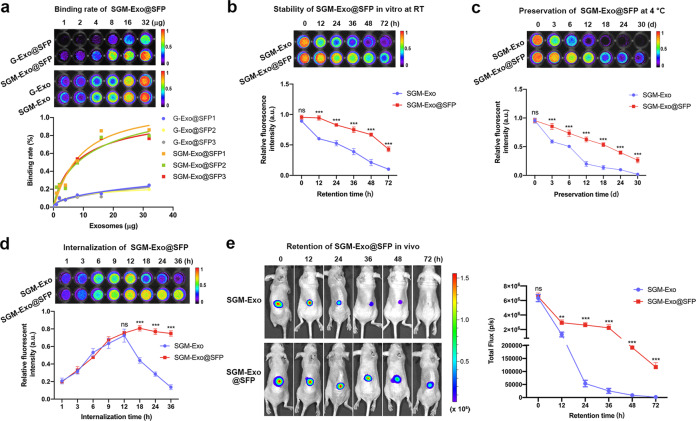
SFP enhanced the binding rate, release sustainability, and stability of SGM-Exos. **a** The binding rate of SGM-Exos with SFP was significantly higher than that of G-Exos (*n* = 6/group). **b** SFP enhanced the stability of SGM-Exos at RT (*n* = 8/group). **c** SFP enhanced the stability of SGM-Exos at 4 °C (*n* = 8/group). **d** Internalization of SGM-Exos released from SFP by HaCaT cells (*n* = 8/group). **e** Retention of SGM-Exo and SGM-Exo@SFP in vivo monitored by Gaussia luciferase (Gluc) activity (*n* = 6/group). The signal activity was expressed as photons/s/cm^2^/steradian (sr). Data represent the mean ± S.D. of three different experiments (ns: no significance, **p < 0.01, ***p < 0.001)

During the process of exosome biogenesis, Gaussia luciferase is expressed into exosomal membrane to mark exosomes. Then we compared the binding rates of SGM-Exos and G-Exos with SFP by measuring Gluc signals. It was found that the binding rate of SGM-Exos with SFP was increased as well as G-Exos with the increasing exosome concentration. At the same exosome concentration, the binding rate of SGM-Exos with SFP was three times higher than that of G-Exos (Fig. 3a). Next, we investigated the stability of exosomes under two storage temperatures (RT and 4 °C) and found that the Gluc signals lasted for a longer time in SGM-Exo@SFP group compared with that in SGM-Exo group whether at RT or 4 °C, indicating that SFP could enhance the stability of SGM-Exos (Fig. 3b and c). Furthermore, the basic expression of miR146a in SGM-Exo@SFP group was also higher than that in SGM-Exo group, showing the protective effects of SGM-Exo@SFP on miR146a (Supplementary Fig. S3). Through internalization experiment of HaCaT cells, we confirmed that SGM-Exo@SFP could continuously provide SGM-Exo for receptor cells in vitro (Fig. 3d). Finally, we observed the sustained release of SGM-Exos from SFP in vivo. The BLI data revealed that robust Gluc signals of the points were within 12 h in both groups. In comparison with quickly descending signals in the group of SGM-Exo, the Gluc signals in the group of SGM-Exo@SFP could be acquired until 72 h (Fig. 3e). Together, these results indicated that SFBP enhanced the binding rate of SGM-Exos onto SFP and SFP could improve sustained release and stability of SGM-Exos.

### Encapsulation of miR146a and silencing effects

**Fig. 4:**
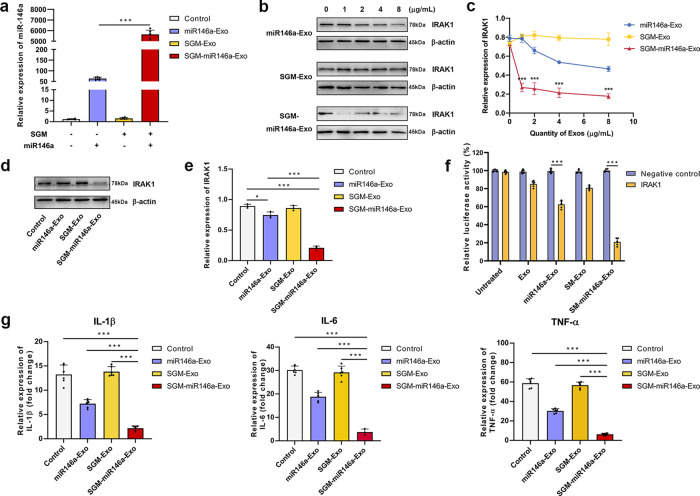
Expression detection of miR146a in engineered exosomes and evaluation of miR146a silencing effects. **a** Expression of miR146a in engineered exosomes assessed by quantitative RT-PCR (*n* = 6/group). **b**, **c** Western blot analysis of IRAK1 expression levels in HaCaT cells treated with gradient concentrations of engineered exosomes. **d**, **e** Western blot analysis of the inhibitory effect of 1 μg/mL engineered exosomes on IRAK1 expression. **f** Dual-luciferase reporter assays of IRAK1 expression in each group. **g** ELISA analysis of inflammatory factors IL-1β, IL-6, and TNF-α. (*n* = 6/group). Data represent the mean ± S.D. of three different experiments (*p < 0.05, ***p < 0.001)

In order to evaluate the loading efficiency of miR146a in SGM-miR146a-Exos, we detected the expression of miR146a in different exosomes especially the engineered exosomes transfected with pac-miR146a-pac and/or SGM protein. In addition to a basic expression of miR146a in PMSC-derived exosomes, overexpression of pac-miR146a-pac notably boosted the miR146a level in miR146a-Exos. Furthermore, pac-miR146a-pac and SGM co-transfection significantly enhanced about ten-folds loading efficiency of miR146a in SGM-miR146a-Exos via pac/MS2 element interaction (Fig. 4a). Additionally, dose-dependent relationship between miR146a expression levels and the amount of SGM-miR146a-Exos, used to treat receptor cells, was also observed (Supplementary Fig. S5).

Next, we detected the biological function of miR146a in SGM-miR146a-Exos to silence the target genes. Interleukin-1 receptor-associated kinase 1(IRAK1) was a potential target of miR146a which identified in recent studies,^26,27^ and the Targetscan (www.targetscan.org/vert_71/) also supported it base on several putative miR146a binding site in the 3’-untranslated regions. To evaluate the silencing effects of miR146a in SGM-miR146a-Exos on IRAK1 expression, HaCaT cells were treated with gradient concentration of G-Exos, miR146a-Exos, SGM-Exos, and SGM-miR146a-Exos, respectively. The results demonstrated that SGM-miR146a-Exos had excellence inhibiting effect on IRAK1 compared with SGM-Exos and miR146a-Exos in transcription and protein levels with a dose-dependent manner. (Fig. 4b and c). At the concentration of 1 μg/mL, IRAK1 expression was slightly decreased in miR146a-Exos group, but significantly downregulated in SGM-miR146a-Exos group (Fig. 4d, e). The dual-luciferase reporter genes Fluc and Rluc were used to evaluate the inhibitory effects of miR146a on the target genes. Here, the exosomes without Gluc fusion protein tags (SM-Exos and SM-miR146a-Exos) were used in the double luciferase assay to avoid the influence on luciferase observation of Fluc and Rluc. The result showed the SM-miR146a-Exos significantly downregulated the fluorescence intensity of IRAK1 (Fig. 4f, Supplementary Fig. S6).

IRAK1 is well known to be an upstream regulator of NF-κB inflammation signaling pathway. Consequently, we monitored cellular supernatant with ELISA, and the inflammatory cytokines interleukin-1β (IL-1β), interleukin-6 (IL-6), and tumor necrosis factor-α (TNF-α) were significantly decreased with treatment of SGM-miR146a-Exos as expected (Fig. 4g). Together these observations demonstrated that we successfully encapsulated miR146a in SGM-miR146a-Exos with high efficiency and miR146a performed favorable biological function.

### Wound healing promoting effects of SGM-miR146-Exos@SFP

**Fig. 5:**
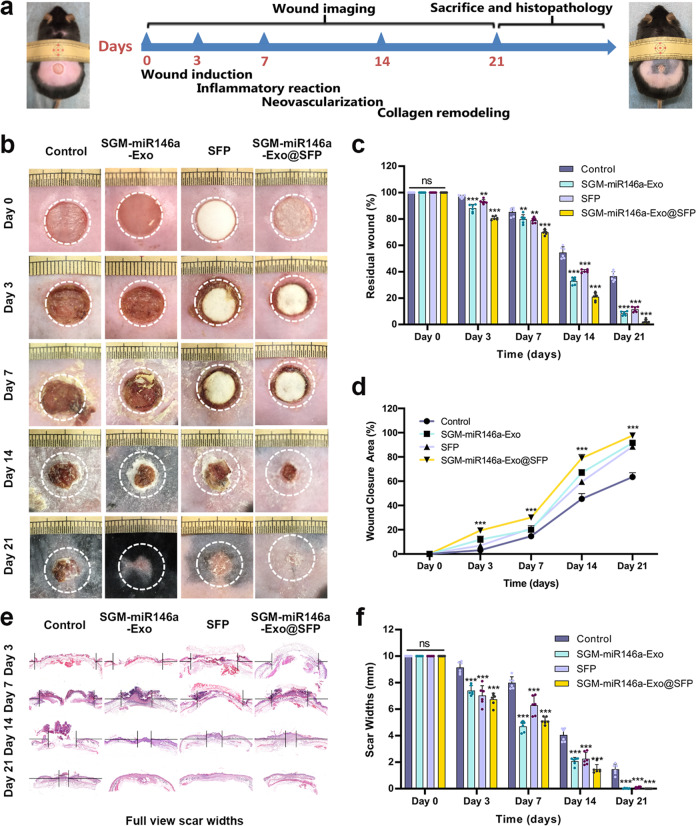
SGM-miR146a-Exo@SFP accelerated wound closure in diabetic mice. **a** Schematic illustration of wound treatment process. **b** Gross view of wounds at days 0, 3, 7, 14, and 21. The white circles represent the original wound areas. **c** Statistical analysis of the residual wound area in each group (*n* = 6/group). **d** Statistical analysis of wound closure area (*n* = 6/group). **e** Representative images of scar widths stained by H&E. **f** Statistical analysis of the scar widths in each group (*n* = 6/group). Data represent the mean ± S.D. (ns: no significance, **p < 0.01, ***p < 0.001)

Subsequently, we investigated whether the SGM-miR146a-Exo@SFP could accelerate diabetic wound healing under in vivo conditions. The experimental procedure has been displayed in Fig. 5a. Full-thickness wounds on the back of BKS-DB (db/db) mice were locally treated with SGM-miR146a-Exos, SFP, or SGM-miR146a-Exo@SFP and untreated wounds were used as control. No death or abnormality was observed in any animal during the postoperative period. Wounds treated with SFP and SGM-miR146a-Exo@SFP were much faster in hemostasis. Gross observation of dorsal wounds indicated that the wound healing process was significantly accelerated by local application of SGM-miR146a-Exos, SFP, or SGM-miR146a-Exo@SFP compared with control group (Fig. 5b). The wound areas were measured at days 0, 3, 7, 14, and 21 after wounding. As shown in Fig. 5c, d, mice treated with SGM-miR146a-Exos, SFP, or SGM-miR146a-Exo@SFP displayed greater wound closure than observed in the control group at days 3, 7, 14, and 21 post-wounding. The fastest healing in SGM-miR146a-Exos, SFP, and SGM-miR146a-Exo@SFP groups occurred between 7 and 14 days. At day 21, the SGM-miR146a-Exo@SFP group wounds were completely healed, but the wound area in control group was still about 40%. Furthermore, the degree of wound healing in SGM-miR146a-Exo@SFP group was significantly better than that in SGM-miR146a-Exos or SFP groups, indicating an additional pro-healing function between SGM-miR146a-Exo and SFP. Consistent with the wound healing, the narrowest scar widths were observed in SGM-miR146a-Exo@SFP group at days 3, 7, and 14 post-wounding (Fig. 5e, f).

### Granulation tissue formation, re-epithelialization, and collagen deposition promoted by SGM-miR146-Exo@SFP

**Fig. 6:**
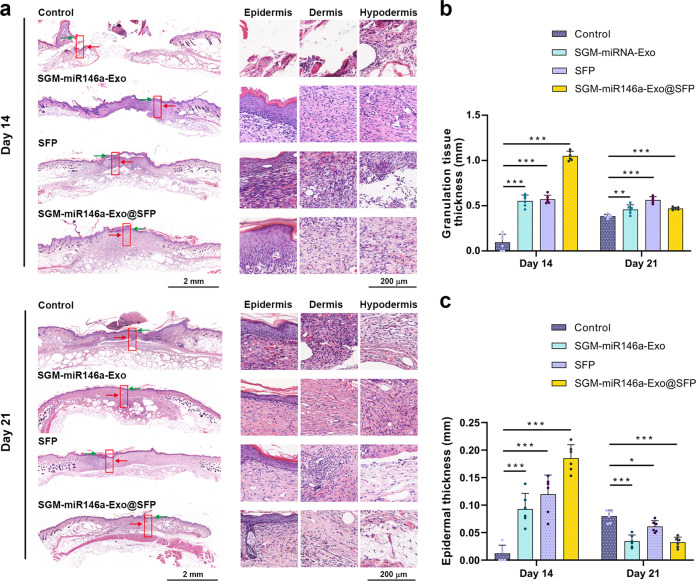
Granulation tissue formation and re-epithelialization promoted by SGM-miR146-Exo@SFP. **a** Representative images of skin tissue section from the wounds on day 14 and day 21 stained by H&E (left, scale bar: 2 mm). High-magnification images showed granulation tissue formation and epithelial tissue closure (right, scale bar: 200 μm). Hair follicles and sebaceous glands could be observed in SGM-miR146a-Exo@SFP-treated wounds at day 21 (bottom right). The extent of epidermis and granulation tissue were marked with green arrows and red arrows respectively. **b**, **c** Statistical analysis of granulation tissue thickness and epidermal thickness in each group (*n* = 6/group). Data represent the mean ± S.D. (*p < 0.05, **p < 0.01, ***p < 0.001)

Histological staining was performed to observe granulation tissue formation and re-epithelialization in repaired tissues. Figure 6a showed representative H & E images of the wounds treated as well as the above methods at day 14 and day 21. The formation of granulation tissue and epidermal tissue was assessed by measuring the thickness of granulation tissue and epidermal tissue. The results showed that SGM-miR146a-Exo@SFP treated wounds displayed a significant increase in the granulation tissue thickness at day 14 in contrast with control, SGM-miR146a-Exos, and SFP groups. But at day 21, the granulation tissue thickness in SGM-miR146a-Exo@SFP group decreased compared with day 14, which was almost equivalent to the level of SGM-miR146a-Exos and SFP groups (Fig. 6a and b).

Rapid re-epithelialization is one of the most important steps in wound healing. Regarding the thickness of neo-epidermis, at day 14, it was thicker in SGM-miR146a-Exos, SFP, and SGM-miR146a-Exo@SFP groups (89.3 ± 17.25 μm, 121.15 ± 19.35 μm, 176.2 ± 14.65 μm, respectively) than in the control group (10.54 ± 9.75 μm), indicating an accelerated re-epithelialization progression. At day 21, the new epithelium of the SGM-miR146a-Exo@SFP group exhibited well-arranged and there were regularly distributed keratinocytes throughout the epithelium, achieving the re-epithelialization. By contrast, the the neo-epidermis morphology of the control group was irregularly arranged and hyperplastic (Fig. 6a and c). More encouragingly, mature skin structures such as hair follicles and sebaceous glands were observed in SGM-miR146a-Exo@SFP treated wounds at day 21 (Fig. 6a).

To observe the effect of SGM-miR146a-Exo@SFP on collagen deposition and remodeling, Masson’s trichrome staining was used to stain collagen fibers. As shown in Supplementary Fig. S7, the increased collagen deposition was observed and the collagen fibers were already normally arranged in the SGM-miR146a-Exo@SFP group at day 14. At day 21, the increased collagen deposition and typical bundle-shaped collagen fibers were still observed in SGM-miR146a-Exo, SFP and SGM-miR146a-Exo@SFP groups, whereas the control groups presented a chaotic outlook with inflammatory cell infiltration.

Together, these results indicated that SGM-miR146a-Exo@SFP enhanced granulation tissue formation, re-epithelialization process, collagen deposition, and even the regeneration of hair follicles and sebaceous glands.

### Anti-inflammation and angiogenesis roles of SGM-miR146a-Exo@SFP

**Fig. 7:**
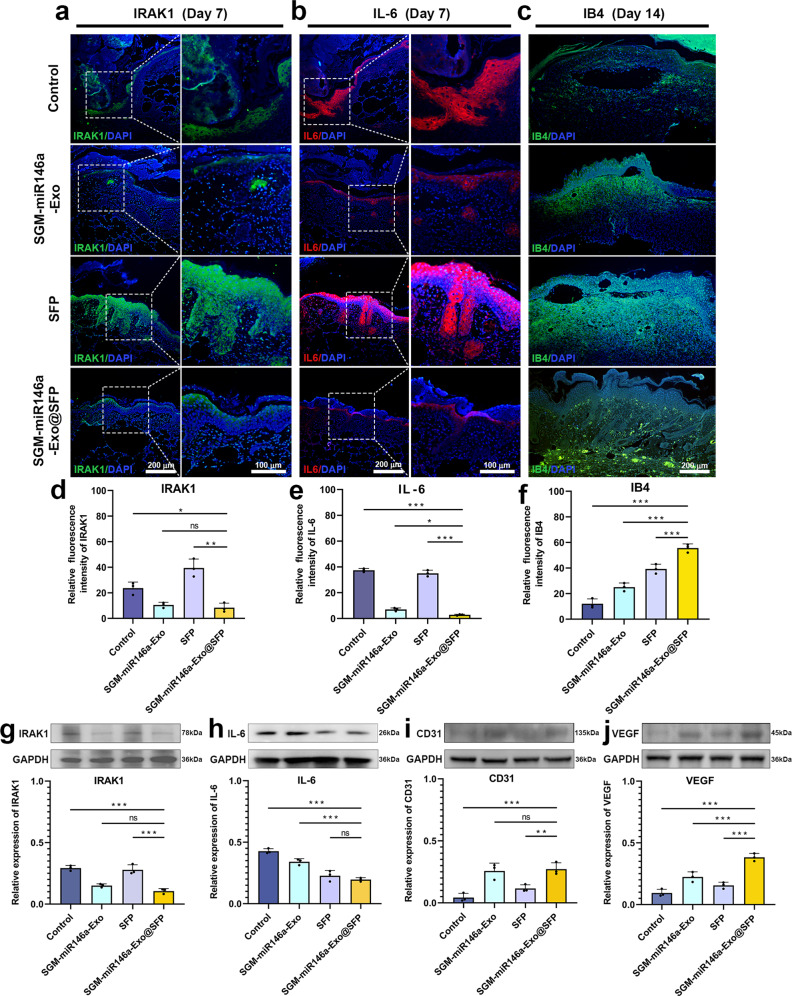
SGM-miR146a-Exo@SFP attenuated inflammation and increased neovascularization of diabetic wounds. **a**–**c** Immunofluorescence of IRAK1, IL-6, and IB4 of the wound tissue (scale bar: 200 μm). **d**–**f** Statistical analyses of IRAK1, IL-6, and IB4 immunofluorescence. **g** The IRAK1 expression was decreased in SGM-miR146a-Exo@SFP group. **h** The IL-6 expression was decreased in SGM-miR146a-Exo@SFP group. **i** The increased CD31 expression in SGM-miR146a-Exo@SFP group. **j** The increased VEGF expression in SGM-miR146a-Exo@SFP group. Data represent the mean ± S.D. of three different experiments (ns: no significance, *p < 0.05, **p < 0.01, ***p < 0.001)

The inflammation phase is a critical process of the wound healing, while the prolonged or exaggerated inflammatory response has been suggested to be the pathogenic mechanism for chronic wounds. Next, we further confirmed the anti-inflammation role of SGM-miR146a-Exo@SFP in wound treatment. IRAK1 and IL-6 were chosen as the indicators for evaluating inflammatory activity and anti-inflammatory efficacy. Consistent with the silencing effects of miR146a in SGM-miR146a-Exos on IRAK1 expression in vitro, IRAK1 expression in vivo was obviously inhibited in SGM-miR146a-Exo or SGM-miR146a-Exo@SFP treated wounds (Fig. 7a, d). IL-6, as downstream gene regulated by IRAK1, is one of representative pro-inflammatory cytokines.^28,29^ As expected, IL-6 expression in the wounds was effectively attenuated in SGM-miR146a-Exo@SFP group compared with control, SGM-miR146a-Exos, and SFP groups. Furthermore, SGM-miR146a-Exo group also had a decreased expression of IL-6 in contrast with control group (Fig. 7b, e). The anti-inflammatory effect was verified at the protein level. The results showed that IRAK1 and IL-6 expressions were apparently decreased in SGM-miR146a-Exo@SFP group (Fig. 7g, h).

During wound healing, new blood vessel formation is crucial for transport of trophic factors to wound sites. We next investigated whether treatment with SGM-miR146a-Exo@SFP could promote angiogenesis in wound sites, thereby enhancing diabetic wound healing. As shown in Fig. 7c, f, isolectin B4 (IB4), a specific marker of angiogenesis,^30,31^ showed increased expression in SGM-miR146a-Exos, SFP, and SGM-miR146a-Exo@SFP groups, indicating enhanced neovascularization compared with control group. Besides, more mature vessels marked with IB4 were observed in SGM-miR146a-Exo@SFP group. CD31 and VEGF expressions detected by western blotting were also increased in SGM-miR146a-Exo@SFP group (Fig. 7i, j). Furthermore, the new blood vessels around the wounds were notably observed in SGM-miR146a-Exo@SFP group on day 14 by the stereo microscope (Supplementary Fig. S8). Collectively, these findings suggested that SGM-miR146a-Exo@SFP could promote angiogenesis and inhibit inflammatory response at wound sites in diabetic mice.

### Mechanism investigation of wound healing promoted by SGM-miR146-Exos@SFP

**Fig. 8:**
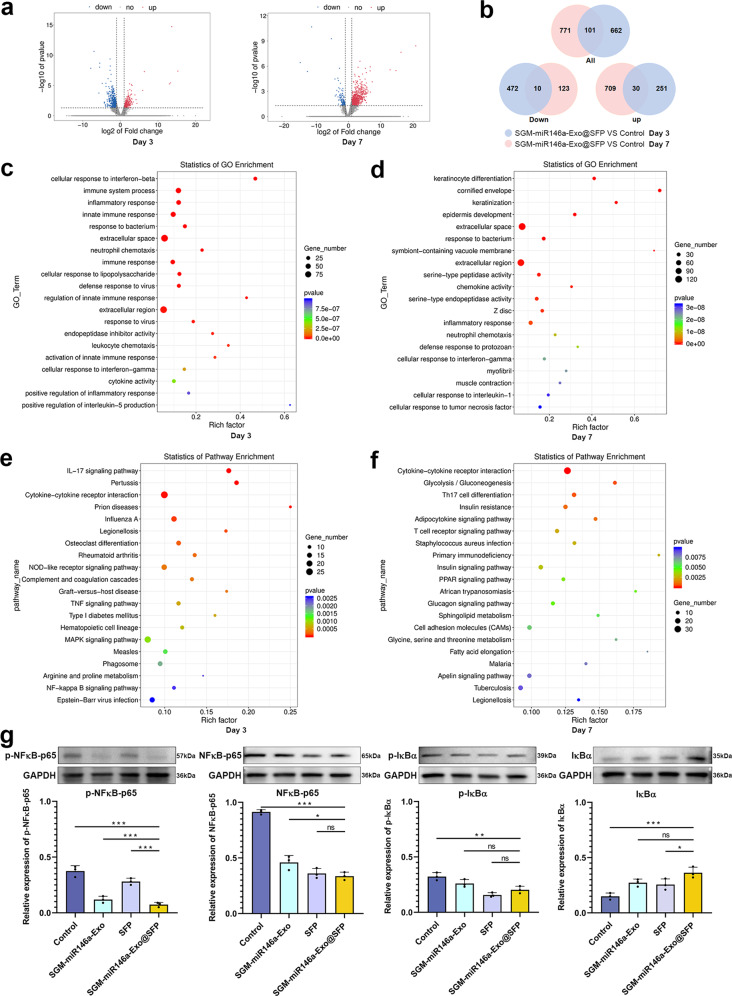
Transcriptomics analysis of the wound tissue and confirmation experiment of the inflammation signaling pathways. **a** Volcano plot of differentially expressed genes (DEGs) at day 3 and day 7. **b** Venn diagram of two overlapping circles representing the overlapped down and up genes at day 3 and day 7. **c** Gene Ontology (GO) enrichment analysis of DEGs. **d** DEGs at day 7 were enriched in skin development and differentiation. **e** KEGG pathway enrichment analysis of DEGs. **f** SGM-miR146a-Exo@SFP upregulated insulin pathway and cell adhesion molecule (CAM) pathway at day 7. **g** The expressions of p-NFκB-p65, NFκB-p65, p-IκBα, and IκBα in wound tissue. Data represent the mean ± S.D. of three different experiments (ns, no significance, *p < 0.05, **p < 0.01, ***p < 0.001)

To get insight into the mechanism of the wound healing process, we performed transcriptomics analysis of wounds treated with or without SGM-miR146a-Exo@SFP at day 3 and day 7 post wounding using high-throughput mRNA sequencing (Supplementary Fig. S9). The differentially expressed genes (DEGs) were defined as having a minimum of two-fold change in expression. Figure 8a showed that the expression profiles of SGM-miR146a-Exo@SFP group were distinct from the profiles of control. We found 482 and 133 genes with significantly decreased transcript levels in SGM-miR146a-Exo@SFP treated wounds compared to the wounds in control group at day 3 and 7 respectively. There were 281 genes that exhibited significantly increased transcript levels in SGM-miR146a-Exo@SFP treated wounds at day 3 and 739 at day 7 (Fig. 8b). These results demonstrate that downregulated DEGs predominated in SGM-miR146a-Exo@SFP group at day 3 probably due to miR146a silencing effects and upregulated DEGs predominated at day 7 probably due to the activation of repair cells in SGM-miR146a-Exo@SFP treated wounds. Top 20 DEGs associated with inflammation and re-epithelialization demonstrating the significant increase or decrease in transcript levels from each time point were respectively presented in Tables S18 and S19. Among the DEGs, we noted that the inflammation-related genes, including IL-1b, IL-1r2, IL-11, IL-19, IL-23a, Fasl, TNF, NF-κB, Tlr4, Tlr6, CXCR2, and CXCR5, were significantly reduced in SGM-miR146a-Exo@SFP group at day 3 (Supplementary Table S17). At day 7, we observed that the genes related to the development and differentiation of epidermis, including Sprr1a, Sprr1b, Sprr2a1, Sprr2a2, Sprr2d, Sprr2e, Sprr2f, Sprr2g, Sprr2h, Sprr2i, Krt10, Krt6a, Cnfn, Pou3f1, Trp63, Egf, Epgn, and Ereg, were greatly increased in SGM-miR146a-Exo@SFP treated wounds (Supplementary Table S18). Through Gene Ontology (GO) enrichment analysis of DEGs, we found that the downregulated DEGs in SGM-miR146a-Exo@SFP group at day 3 were distinctly enriched in “IFN response”, “immune response”, “inflammatory response”, and “innate immunity” (Fig. 8c) and upregulated DEGs at day 7 were enriched in epidermal development and keratinocytes differentiation (Fig. 8d). The statistics of Kyoto Encyclopedia of Genes and Genomes (KEGG) pathway enrichment analysis of DEGs indicated that SGM-miR146a-Exo@SFP downregulated interleukin, TNF, NF-κB signaling pathways at day 3 (Fig. 8e) and upregulated insulin pathway and cell adhesion molecule (CAM) pathway which could be involved in cell development and differentiation^32^ at day 7 (Fig. 8f). The confirmation experiment of the inflammation signaling pathways was performed on the expressions of p-NFκB-p65, NFκB-p65, p-IκBα, and IκBα. The results indicated the expressions of p-NFκB-p65 were obviously decreased and the activation of NFκB-p65 were inhibited in SGM-miR146a-Exo@SFP group. The expression levels of p-IκBα were downregulated and IκBα were reactively upregulated in SGM-miR146a-Exo, SFP, and SGM-miR146a-Exo@SFP group compared with control. (Fig. 8g). Finally, we performed the association analysis of 52 DEGs involved in inflammatory reaction and cellular proliferation and differentiation during wound healing. Supplementary Fig. S10 revealed the interaction relationship among these DEGs, with IL-1b, IL-1r2, NF-κB, Tlr4, and Tlr6 the most common, which could be used as the potential therapeutic targets to promote wound healing.

## Discussion

EVs or exosomes, as natural nano-scale drug carriers, provide novel promising delivery systems for the therapy of diseases by loading effective agents. Recently, various biomaterials are used to extend the retention time of exosomes and control the release of therapeutic agents in vivo, which are expected to become a practically powerful tool for chronic wound treatment.^33^ In the treatment strategy, the loading efficiency of pro-healing agents into exosomes and exosomes onto biomaterials is crucial. In this study, we succeeded in engineering the exosomes for efficient miR146a loading and for the special attachment to the surface of SFP by constructing a fusion protein in which the exosomal membrane protein Lactadherin was fused with RNA binding protein and SFBP. In vitro, SGM-miR146a-Exos exhibited excellent inhibiting effect on NF-κB signaling pathway by miR146a targeting IRAK1, thereby downregulating the expressions of a large number of inflammatory cytokines. In vivo, SGM-miR146a-Exo@SFP significantly enhanced the wound repair associated with anti-inflammatory, collagen deposition, and neovascularization. Supplementary Fig. S11 showed the schematic illustration of SGM-miR146a-Exos released from SFP promoting diabetic wound healing.

MiRNAs, as possible therapeutic agents, need an effective delivery system to transfer into target cells. MS2 is a single-stranded RNA bacteriophage and the RNA is composed of 3569 nucleotides encoding four genes including the maturation protein, the capsid protein, the lysisprotein, and the replicase protein. The RNA genome package by capsid protein starts from the binding between the capsid protein dimer and a specific 19-nucleotide stem-loop region (pac site). Recently, the binding specificity of MS2 capsid protein for RNA recognition have been applied for developing virus-like particles for drug delivery. In our experiments, we fused Lactadherin (a membrane-associated protein enriched in exosomes) with MS2 capsid protein at the C terminal to recognize the designed miR146a which have two pac sites at flanks. During the process of exosome biogenesis, the fused Lactadherin-MS2 successfully enriched miR146a into exosomes when miR146a was excessively expressed in PMSCs. Compared to miR146a-Exos without the help of MS2 pac/capsid protein element interaction, a ten-fold increased loading efficiency of miR146a was observed in MS2-miR146a-Exos.

Even though there are so many researches about effects of EVs on wound treatment, the administration and retention of EVs in wounds still face challenges. Application of biomaterials provides sustainable release and even distribution of EVs on wound surface.^34,35^ Silk is a natural high polymer fiber protein, containing 70–80% silk fibroin with good mechanical, physical, and chemical properties. In our experiment, SFP was served as exosome-laden scaffolds after evaluation of biological safety in vivo. To enhance the loading efficiency of exosomes onto SFP, we inserted SFBP between SP and C1C2 domains of exosomal membrane protein Lactadherin. With the help of SFBP presented on the exosomal membrane, the SF-affinity exosomes distributed more densely on SFP compared with the natural exosomes without the capacity of SF-affinity. Additionally, we also fused Lactadherin with Gluc following SFBP as a good agent for exosome labeling.

Chronic diabetic wounds occur frequently in diabetic patients such as diabetic foot and diabetic ulcer. Persistent inflammation and vascular dysfunction are the main causes of diabetic wound unhealing, so suppressing inflammation and promoting angiogenesis can help tackle the root of chronic wound healing problem.^36,37^ In this research, miRNA-146a as an anti-inflammatory regulator was encapsulated in exosomes and then the exosomes were loaded onto SFP. With above efficient miRNA delivery system, we created a new wound dressing named SGM-miR146a-Exo@SFP. In vivo experiments showed that SGM-miR146-Exos@SFP accelerated diabetic wound healing by inhibiting inflammatory response and promoting angiogenesis companied with enhanced granulation tissue formation, re-epithelialization, and collagen deposition. Consistent with our results, Zeng et al.^38^ and Li et al.^39^ also reported the function of miR146 to repress inflammatory response in acute lung injury and induce angiogenesis in osteoarthritis cartilage.

We next investigated the mechanism responsible for the anti-inflammatory and angiogenesis function of miR146 during wound healing. MiR146a has been predicted to target TNF receptor-related factor 6 (TRAF6), IRAK1, and Smad4. Researchers found that miR146a inhibited the expression of TRAF6 and IRAK1 and blocked the activation of TLRs/NF-κB signaling pathway, thereby reducing the release of pulmonary inflammatory factors.^38^ In our experiment, miR146a inhibited the expression of IRAK1, TNF-α, IL-1β, and IL-6 in vitro and the expression of IRAK1, IL-6, and p-NFκB-p65 in vivo. In terms of angiogenesis function, we found that miR146 enhanced the expression of IB4, CD31, and VEGF. Consistent with our results, Li et al.^39^ reported that miR146a upregulated VEGF expression through targeted inhibition of Smad4, contributing to the pathogenesis of osteoarthritis.

Collectively, we generated engineered exosomes SGM-miR146a-Exo by fusing Lactadherin with SFBP between SP and C1C2 domains to attach the surface of SFP, with Gluc following SFBP to monitor exosomes in real-time, and with MS2 at the C terminal to load designed miR146a which have two pac sites at flanks. SGM-miR146a-Exo@SFP created as a new wound dressing was proven to inhibit inflammation and promote angiogenesis, thereby accelerating wound healing. Consequently, this study not only provides an efficient approach to enrich miRNAs into exosomes, but also develops a practical exosome@biomaterial-based drug delivery platform for miRNAs to treat diabetic wounds.

## Materials and methods

### Ethics statement

The animal experiments were approved by the medical ethics committee of Chinese PLA General Hospital.

### Phage display for SFBP

The SFBP was selected through screening the 12-phage peptide library and validation by ELISA. The biopanning of SFBP schematic was shown in Supplementary Fig. S1. The detailed screening and preparation methods of SFBP were provided in the Supplementary Material.

### Preparation and identification of exosomes

Exosomes derived from PMSC (Exo), G-PMSC (G-Exo), SGM-PMSC (SGM-Exo), miR146a-PMSC (miR146a-Exo), and SGM-miR146a-PMSC (SGM-miR146a-Exo) were prepared as the methods described in the Supplementary Material. Exo and G-Exo were used as control. All the exosomes were summarized in a Table (Supplementary Table S17). Exosome morphologies were observed using transmission electron microscopy (TEM). The size distribution of exosomes was measured by using Nanosizer technology (Malvern Instruments, Malvern, U.K.). Expression of the exosomal characteristic markers CD9 (1:500), CD63 (1:1,000), TSG101 (1:500), and Calnexin (1:500) (CST, MA, USA) were analyzed by Western blotting.

### Preparation of SFP encapsulating engineered exosomes

The silk fibroin protein-based porous materials were prepared as the methods in the Supplementary Material. The silk fibroin protein porous materials were prepared into patches of the wound size. 100 μg of exosome solution (50 wt%) was added to the SFP, followed by incubating at 37 °C for 10 min for exosome attaching to the SFP. The uncombined exosomes were washed away from SFP with deionized water. Finally, SGM-Exo loaded SFP (SGM-Exos@SFP) was obtained through the above process. The control SFP was infiltrated with PBS for detection.

### Release and internalization of Gluc-labeled exosomes

The total protein of exosomes was monitored by Gaussia luciferase (Gluc) activity using an exposure apparatus. The release ratio of exosomes was tested in the presence of the Gluc substrate water-soluble coelenterazine (GeneCopoeia). Briefly, the Gluc-labeled exosomes (G-Exos) isolated from PMSCs, which transduced with Gluc fusion protein, were used as the control exosome in binding rate analysis. 100 μg exosomes were added in a patch of 10-mm diameter, following combination with each other in 48-well plate at 37 °C for 10 min. Then, adding 200 μL PBS in each well to submerge the SFP. After incubating in a 37 °C incubator for differential time, the supernatant PBS was collected and moved to another 48 wells plate for BLI analysis. The amounts of leaching exosomes were measured via Gluc signals according to the trend of linear dependent relationship between the quantity of exosomes and Gluc activity. The internalization of G-Exos was analyzed in HaCaT cells. HaCaT cells were seeded at 5 × 10^4^ cells per well in a 24-well plate one day prior to adding SGM-Exos (100 μg/mL) and SGM-Exo@SFP, then incubated for 1, 3, 6, 9, 12, 18, 24, and 36 h. After washing by PBS twice, the internalization of exosomes was analyzed by luminescence imaging system.

### BLI analysis

The retention of SGM-Exo and SGM-Exo@SFP were monitored by Gaussia luciferase (Gluc) activity. In the vivo experiments, 100 μg SGM-Exos in a volume of 100 μL were loaded in SFP, and then the SGM-Exo@SFP was subcutaneously implanted in the back of nude mice. 100 μg SGM-Exo solution without SFP was used as control. At the indicated time points (0, 12, 24, 36, 48, and 72 h), water-soluble coelenterazine (5 mg/kg, Nanolight Technology) was injected subcutaneously and the animals were imaged immediately using IVIS Lumina Imaging System. The signal activity of BLI was expressed as photons/s/cm^2^/steradian (sr).

### Dual-luciferase reporter assay

Renilla luciferase (Rluc) was used as an internal control. The firefly luciferin (Fluc) was used as the substrate to detect the expression of IRAK1. Luciferase catalyzed the oxidation of luciferin to oxyluciferin with the participation of Mg^2+^ and O_2_. In the oxidation process of luciferin, bioluminomescence occurred. Then, in subsequent experiments, the bioluminescence of Fluc was terminated by the addition of coelenterazine, the substrate of Rluc that inhibited the luciferase catalysis of Fluc. Bioluminescence occurred through the catalytic oxidation of luciferase to coelenteramide. After 2 min, the bioluminescence was measured by a chemiluminescence instrument. The BLI signals were all measured by average radiance from regions of interest (ROI). In the BLI experiments performed in plates, the ROIs were merged with the wells in each group.

### Diabetic wound model treatment and analysis

The detailed methods of diabetic wound model treatment and analysis were provided in the Supplementary Materials and Methods.

### Statistical analysis

All results presented are from at least three independent experiments for each condition. Data are expressed as mean ± standard deviation (S.D.). One-way or two-way analysis of variance was used to determine the level of significance using GraphPad Prism 9.2 software. Differences were considered statistically significant at *p* < 0.05.

## Supplementary information

Supplementary Materials for MiR146a-loaded engineered exosomes released from silk fibroin patch promote diabetic wound healing by targeting IRAK1

## Data Availability

The RNA-seq data generated during this study have been deposited in the NCBI’s Gene Expression Omnibus (GSE217981). Other data that support the findings of this study are available from the corresponding author upon reasonable request.
