# Supplementary material for: MiR146a-loaded engineered exosomes released from silk fibroin patch promote diabetic wound healing by targeting IRAK1
Source: Signal Transduct Target Ther. 2023 Feb 13;8:62. doi: 10.1038/s41392-022-01263-w (PMC9922687; doi:10.1038/s41392-022-01263-w)
Supplement: Supplementary file 1 — Supplementary Materials for MiR146a-loaded engineered exosomes released from silk fibroin patch promote diabetic wound healing by targeting IRAK1 [file 41392_2022_1263_MOESM1_ESM.docx]

Supplementary Materials for

MiR146a-loaded engineered exosomes released from silk fibroin patch promote diabetic wound healing by targeting IRAK1

Qiankun Li^1,2,a^, Wenzhi Hu^2,a^, Qilin Huang^2^, Jie Yang^2^, Bingmin Li^2,3^, Kui Ma^2,4,5^, Qian Wei^2^, Yaxi Wang^2^, Jianlong Su^2,6^, Mengli Sun^2^, Shengnan Cui^2^, Rungong Yang^1^, Haihong Li^7,*^, Xiaobing Fu^1,2,4,5,*^, and Cuiping Zhang^2,4,5,*^

Correspondence to: [zcp666666@sohu.com](mailto:zcp666666@sohu.com); [fuxiaobing@vip.sina.com](mailto:fuxiaobing@vip.sina.com); [lihaihong1051@126.com](mailto:lihaihong1051@126.com)

**This PDF file includes:**

Materials and Methods

Figure S1 to S11

Table S1 to S19

Materials and Methods

**Cell isolation and culture.** The human placenta-derived mesenchymal stem cells (PMSCs) were obtained from Health & Biotech and identified as described previously.^1^ PMSCs were cultured in the mixed medium of high glucose Dulbecco's Modified Eagle Medium (DMEM) and DMEM/F12 medium (Gibco, CA, USA) at a ratio of 1:1 with 10% exosome-free FBS (SBI, CA, USA) and 100 U/mL penicillin-streptomycin (PS) (Gibco). The PMSCs used in the subsequent experiments were between passages 3 and 8. Human immortalized keratinocytes (HaCaT cells) were obtained from ATCC Cell Lines Service (Manassas, VA, USA). The medium for HaCaT cells was Epilife^TM^ medium (Gibco). 293T cells were maintained in DMEM with 100 U/mL penicillin-streptomycin (Gibco) and 2 mM L-glutamine (Gibco). All cells were cultured in a humidified atmosphere of 5% CO_2_ at 37 °C.

**Phage display for silk fibroin binding peptide (SFBP).** The SFBP was selected through screening the 12-phage peptide library and validation by ELISA. Briefly, plates were coated with 10 mg/mL antigen (SF) incubate at 60 °C for membrane forming. The library phage was inputted into the plates and incubated for 1.5 h at 37 °C. Then the diluted eluate phage and blent 10 μL dilution with 180 μL E. coli TG1. Precipitation was centrifuged and suspended with 1 mL PBS. The supernatant was transferred to a sterile tube, which is the amplified phages and could be used in the next biopanning. After 3-4 rounds of biopanning, the phage supernatant was added to the wells for polyclonal and monoclonal phage ELISA. Plates were coated with 2 mg/mL antigen incubate at 60 °C for membrane forming. Diluted anti-M13-HRP antibody (1:5000) was added 100 μL in per well and incubated at 37 °C for 1 h. 100 μL TMB per well was added and incubated at room temperature and then 100 μL 2M HCl per well was added. Plates were read using a microplate reader set at 450nm-620nm. Then the superior clones were detected again by ELISA for verification. Then sequence analysis of the high-specific clones was performed. The biopanning of silk fibroin binding protein schematic was showed in Fig. S1.

**Plasmids and lentivirus-based gene** **transduction.** Human Gaussia luciferase fused with lactadherin (Gluc-lactadherin) were prepared as previously reported^2^ and used in this study to indicate the exosome activity. The Gluc-lactadherin lentiviral plasmid (G-pLV) was constructed to be used for plasmids and lentivirus-based gene transduction**.** Then the sequences of Gluc-lactadherin were inserted into the fragment of C1C2 to construct the SFBP-Gluc-MS2 (SGM) fusion protein and the SGM lentiviral plasmid (SGM-pLV) were constructed. Oligonucleotide P146, which encodes pre-miR146a and pac sites, was chemically synthesized (Sangon Biotech, Shanghai, China), and then inserted into plasmid pMS to construct the recombinant pac-pre-miR146a-pac (miR146a) lentiviral plasmid (miR146a-pLV). The precursor of miR-146a (pre-miR146a) and two C-5 variant pac sites (pac), which were upstream and downstream of pre-miR146a, were designed to be loaded into the MS2 of SGM lentiviral plasmid (SGM-miRNA146a-pLV). A similar strategy was used to construct a control lentiviral vector, pac-pre-miRNC-pac in this study. These recombinant lentiviral plasmids were transfected into 293T cells and the corresponding lentiviral (Lv) particles were packaged. The PMSCs were respectively infected with G-Lv, SGM-Lv, miR146a-Lv and SGM-miR46a-Lv, and then selected by the medium containing 1 μg/mL puromycin (Sigma, MO, USA). Finally, the transduced cells G-PMSCs, SGM-PMSCs, miRNA146a-PMSCs and SGM-miR146a-PMSCs were obtained.

**Isolation and identification of exosomes.** After PMSCs reached approximately 50%–60% confluence, the supernatant was discarded, and the cells were washed with PBS. Then the cells were incubated in high glucose DMEM and DMEM/F12 medium at a ratio of 1:1 with 10% exosome-free FBS and 100 U/mL PS for 48 h. The exosomes were isolated from the conditioned medium of PMSCs as follow. Briefly, the CM was centrifuged at 300 g for 15 min, followed by 2,000 g for 15 min to remove dead cells and cellular debris. Then the supernatants were filtered through a 0.22 mm filter (Merck Millipore) to further remove cellular debris in the CM. The filtered solution was centrifuged at 4,000 g until the volume in the upper compartment was concentrated to approximately 200 mL in a 15-mL Amicon Ultra-15 Centrifugal Filter Unit of 100,000 daltion (Da) molecular weight cut-off (Merck Millipore). The ultrafiltration liquid was washed with PBS, and ultrafiltration was repeated three times at 4,000 g to 200 μL. For further purification, the liquid was ultracentrifuged at 100,000 g for 1 h using a sterile Ultra-Clear tube (Beckman Coulter, Brea, CA, USA) with a 30% sucrose-D2O cushion. The pellets were resuspended in 15 mL of PBS and centrifuged at 4,000 g to approximately 200 μL. All procedures were performed at 4 °C. Exosomes derived from PMSCs (Exo), G-PMSCs (G-Exo), SGM-PMSCs (SGM-Exos), miR146a-PMSCs (miR146a-Exos) and SGM-miR146a-PMSCs (SGM-miR146a-Exos) were isolated as above. The Exo derived from untreated PMSC was the control group, and the G-Exo derived from G-PMSC containing Gaussia luciferase was also used as control in the exosome activity assay. Exosome morphologies were observed using transmission electron microscopy (TEM). The size distribution of exosomes was measured by using Nanosizer technology (Malvern Instruments, Malvern, U.K.). Expression of the exosomal characteristic markers CD9 (1:500), CD63 (1:1,000), TSG101 (1:500) and Calnexin (1:500) (CST, MA, USA) were analyzed by Western blotting.

**Preparation of SFP encapsulating engineered exosomes**. The raw silk was immersed in sodium carbonate solution with a concentration of 0.75 mol/L. The silk was degummed at 85 °C for 45 min, then washed with deionized water and repeated for 5 times to obtain the degummed silk. After drying for 4 h at 60 °C, 9.3 mol/L lithium bromide solution was added to the degummed silk. the degummed silk was dissolved in lithium bromide solution at 60 °C for 4 h. After cooling, the silk fibroin solution was put into a dialysis bag with molecular weight cut off 3,500 Da and dialysed in deionized water for 48 h. During the period, the water was changed every 2 h. After dialysis, the aqueous solution of silk fibroin was centrifuged at 9000 r/min for 2 times. The undissolved precipitate was removed to obtain an aqueous solution of recycled silk protein with a concentration of 6-9%. Higher concentration of silk fibroin protein solution could be obtained by PEG concentration. 7% silk fibroin solution, glycerin (Gly) and PEG400 were mixed with 10:5:3 mass ratio to get a mixed solution. The mixed solution was molded at -80 °C in culture dish. And then the moisture was removed from the molding by vacuum freeze dryer for 48 h. Finally, the silk fibroin protein-based porous materials with β-folding structure were obtained. Then the silk fibroin protein porous materials were prepared into patches of the wound size. 100 μg of exosome solution (50 wt%) was added to the SF patches, followed by incubating in 37 °C for 10 min, the exosomes could attach to SF. Finally, the product, SF affinity exosomes derived from SGM-146a-PMSCs loaded on SFP (SGM-146a-Exos@SFP), was washed with deionized water. Control SF patch was prepared under the same conditions, with the addition of PBS alone instead of exosomes.

**Characterization of Materials.** In order to characterize the morphology and chemical structure properties of SF patch and encapsulated exosome SF patch, the samples were fixed with 2.5% Glutaraldehyde for analyzation. The samples were loaded on top of conductive tapes mounted on SEM sample stubs and sputter-coated with gold for 60 s using gold sputter coating equipment (Cressington Scientific Instruments, Watford, UK). The SF patch and encapsulated exosome SF patch, were detected by Fourier transform IR (FTIR) spectrometry (Vector22; Bruker Daltonics, MA, USA) using the KBr pellet technique.

**Biosafety evaluation of the silk fibroin patch.** HaCaT cells were used for the cytotoxicity test of silk fibroin material in vitro. In the experiment, different proportion of SFP immersion solution were added to the cell culture medium. And different proportion of PBS solution were added to the cell culture medium as control group. For biosafety test in vivo, Balb/c mice were used as experimental animals**.** The SFP group was developed by multiple intraperitoneal injection of 25% SFP immersion solution every two days for 6 weeks. The toxicosis (Tox) group, which used as the positive control, was obtained by multiple intraperitoneal injection of 0.5% CCl4 solution every two days for 6 weeks. The normal saline (NS) group was developed by the above method as control. The liver and kidney, which are important organs for toxin metabolism, were used for biological toxicity analysis in vivo. The blood samples were collected from the mice in different groups for biochemistry. The hepatorenal function indexes were detected to analyse the biological toxicity of SFP to vital internal organs. Moreover, the pathologic changes of the liver and kidney tissue were observed by H&E and Masson staining.

**Release and internalization of Gluc-labeled exosomes.** The total protein of exosomes was monitored by Gaussia luciferase (Gluc) activity using an exposure apparatus. The release ratio of exosomes was tested in the presence of water-soluble coelenterazine (GeneCopoeia). Briefly, the Gluc-labeled exosomes (G-Exos) isolated from PMSCs, which transduced with Gluc fusion protein, were used as the control exosome in binding rate analysis. 100 μg exosomes were added in a patch of 10-mm diameter, following combination with each other in 48-well plate at 37 °C for 10 min. Then, adding 200 μL PBS in each well to submerge the SFP. After incubating in a 37 °C incubator for differential time, the supernatant PBS was collected and moved to another 48 wells plate for BLI analysis. The amounts of leaching exosomes were measured via Gluc signals according to the trend of linear dependent relationship between the quantity of exosomes and Gluc activity. The internalization of G-Exos was analyzed in HaCaT cells. HaCaT cells were seeded at 5 × 10^4^ cells per well in a 24-well plate one day prior to adding SGM-Exos (100 μg/mL) and SGM-Exo@SFP, then incubated for 1, 3, 6, 9, 12, 18, 24 and 36 h. After washing by PBS twice, the internalization of exosomes was analyzed by luminescence imaging system.

**In vivo bioluminescence imaging analysis**. The retention of SGM-Exo and SGM-Exo@SFP were monitored by Gaussia luciferase (Gluc) activity. In the vivo experiments, 100 μg SGM-Exos in a volume of 100 μL were loaded in SFP, and then the SGM-Exo@SFP was subcutaneously implanted in the back of nude mice. 100 μg SGM-Exos solution without SFP were used as control group. After treatment for 0, 12, 24, 36, 48, and 72 h, the mice were imaged using IVIS Lumina Imaging System. At the indicated time points, 0.1 μg/mL water-soluble coelenterazine (5 mg/kg) was injected subcutaneously for luminescence measurement. The signal activity of BLI was expressed as photons/s/cm2/steradian (sr).

**Isolation of miRNA from exosomes and qPCR.** The miRNA was extracted from exosomes of four genotype (Exos, miR146a-Exos, SGM-Exos and SGM-miR146a-Exos), by using a miRcute miRNA Isolation Kit (Tiangen, Beijing, CN), following the manufacturer’s instructions. Synthesis of cDNA was carried out using the miRcute Plus miRNA First-strand cDNA Kit (Tiangen) according to the manufacturer’s instructions. Samples of the cDNA were used as a template for quantitative PCR (qPCR) using the miRcute plus miRNA qPCR kit (SYBR Green) (Tiangen). Reactions were set up to a total volume of 20 μL according to the product protocol and performed on the Stratagene MX3005P qPCR system (Agilent Technologies). Each reaction was set up in triplicate for the target gene 146a under test. Triplicate reactions were also set up with an identical amount of template using primers designed against GAPDH (glyceraldehyde-3-phosphate dehydrogenase) as a housekeeping gene reference standard for normalization of the results. For all qPCR assays, the efficiency of the different primer sets was tested by establishing a standard curve using serial dilutions of a cDNA pool made by combining samples of all the templates used in each experiment. MxPro qPCR Software was used to analyse the dissociation and amplification curves of every experiment and to obtain the threshold cycle values (Ct). Each group has more than 3 samples. Data were then analyzed using Microsoft Excel for quantitation of target gene relative to the reference standard using the 2-ΔΔCT method. T-test analysis were performed on the 2-ΔΔCT values for the samples.

**Western blotting analysis.** HaCaT cells were treated with Exos, miR146a-Exos, SGM-Exos and SGM-miR146a-Exos, respectively. Treated HaCaT cells were lysed by RIPA buffer (Invitrogen) containing protease and phosphatase inhibitors. Protein concentrations were measured by Bicin-choninic acid (BCA) Protein Assay Kit (Solarbio). Equal amounts of heat-denatured protein samples (20 μg) were separated by 12-15% SDS-PAGE gels and then transferred onto PVDF membrane. The transferred protein membranes were blocked with 5% non-fat dried milk for 1 h, followed by incubation with specific primary antibody IRAK1 (CST, MA, USA) at 4 °C overnight, and secondary antibodies for 2 h. GADPH (R&D Systems, Wiesbaden, Germany) was used for internal loading control. The blots were finally detected by chemiluminescence. The protein expression analysis of molecules associated with inflammation and blood vessels in wound tissue were performed with the primary antibody p- NFκB-p65, NFκB-p65, p-IκBα, IκBα, IL-6, CD31, VEGF (CST, MA, USA) and the second antibody Goat Anti-Rabbit/Mouse IgG (TransGen, BJ, CN).

**Dual-luciferase reporter assay.** Renilla luciferase (Rluc) is usually used as an internal control. The firefly luciferin (Fluc) was used as the substrate to detect the expression of IRAK1. Luciferase catalyzes the oxidation of luciferin to oxyluciferin with the participation of Mg^2+^ and O_2_. In the oxidation process of luciferin, bioluminomescence occurs. Then, in subsequent experiments, the bioluminescence of Fluc was terminated by the addition of coelenterazine, the substrate of Rluc that inhibit the Luciferase catalysis of Fluc. Bioluminescence occurs through the catalytic oxidation of luciferase to coelenteramide. After 2 min, the bioluminescence was measured by a chemiluminescence instrument. The BLI signals were all measured by average radiance from regions of interest (ROI). In the BLI experiments which performed in plates, the ROIs were merged with the wells in each group.

**The expression levels of inflammation-related cytokines detected by ELISA**. After treated with exosomes for 3 days, the cell culture mediums were collected from 6-well plates and centrifuged at 4 °C, 3 000 r/min for 15 min. The expression levels of IL-1β, IL-6 and TNF-α in the supernatant of each group were detected by Enzyme-linked Immuno Sorbent Assay (ELISA) kit according to the operation instructions. After colouration, the absorbances were measured at 450 nm by enzyme plate analyzer to establish a standard curve. The concentrations of IL-1β, IL-6 and TNF-α in the samples were calculated by standard curve.

**Diabetic mouse skin wound model and treatment.** All experimental protocols were approved by the Animal Research Committee of Chinese PLA General Hospital. The BKS-DB (db/db) mice were spontaneous type 2 diabetes BKS-Leprem2Cd479/Nju mice and obtained from Nanjing biomedical research institute of Nanjing University. The db/db mice were constructed the mutation of leptin receptor (Lepr) gene by CRISPR/Cas9 technology on a C57BLKS/JNju background for the damage on function of diabetes gene (db). The db/db mice have obvious characteristics of type 2 diabetes, such as hyperglycemia, hyperlipidemia and hyperinsulinemia. The db/db mouse always maintains fasting blood glucose and body mass at a high level, which is an ideal animal model for experimental studies of type 2 diabetes and its complications. The 8-10 weeks male mice were used in this study. All animal experiments complied with the Animal Research: Reporting of In Vivo Experiments guidelines. All experimental protocols were approved by the Animal Research Committee of Chinese PLA General Hospital. The diabetic mice were divided into four groups: control (untreated), SGM-miR146a-Exo (treated with SGM-miR146a-Exo alone), SFP (treated with SFP alone), SGM-miR146a-Exo@SFP (treated with SGM-miR146a-Exo@SFP), and their wounds were observed every day after injury. Eight-week-old BKS mice were acclimatized for 1 week. Wounds were made on the backs of db/db mice. Animals were anesthetized by i.p. injection of 50 mg/kg pentobarbital sodium. Standardized full-thickness skin wounds (10 mm in diameter) were created with biopsy punches (10 mm) by removing epidermis and dermis along with the panniculus carnosus. 10 mm diameter SGM-miR146a-Exo, SFP and SGM-miR146a-Exo@SFP scaffolds were applied on the wound beds, and the wounds were untreated in control groups. After surgery, the animals were caged separately and monitored daily. Digital photographs of the wounds were captured on days 0, 3, 7, 14, 21 after surgery by using a D750 camera (Nikon, Tokyo, Japan) at an equal distance and a calibration scale on the side. The wound area was measured using Image J software (NIH, Bethesda, MD, USA). Wound-size reduction was determined by using the following formula: wound-size reduction (%) = (A0-At)/A0×100, where A0 is the initial wound area (at t = 0) and At is the wound area at day 3, 7, 14 or 21 after surgery. Scar width was measured as the distance between the two ends of the long axis of the wound.

**Histochemical and immunofluorescence staining.** Six wounds per group were studied by histopathological analysis on days 3, 7, 14 and 21 after surgery. The animals were euthanized by cervical dislocation, to harvest skin and muscle samples from the full-thickness defect wound on the back of mice. The wound tissue was excised along with the surrounding tissue and fixed in 4% paraformaldehyde overnight. The excised skin was then dehydrated through a graded series of ethanol, embedded in paraffin, and sectioned perpendicularly to the wound surface into 5-mm thick sections. The sections were stained by using hematoxylin and eosin (H&E) for histological observation. Masson’s trichrome was used to determine the degree of collagen maturity. For histology, stained slides were imaged with a Leica DM 2500 microscope. The length of the neo-epithelium was determined by using a previously described procedure.^3^ Immunofluorescence (IF) staining of the sections were performed to reveal the inflammatory response and the progress of angiogenesis during wound healing. IF staining of IB4 (1: 200; Invitrogen) with bioluminomescence, a marker of endothelial cells, were performed to show the angiogenesis. IF staining for IL-6 (1:50-200; CST) and IRAK1 (1:50-200; CST) were performed to reveal the inflammatory response of the wound tissue. Tissue sections were rehydrated and washed with PBS. For antigen retrieval, tissue sections were treated with preheated antigen retrieval buffer (sodium citrate buffer) to 95 °C for 10 min, followed by permeabilization with 0.1% Triton X-100 for 10 min. After blocked in 10% goat serum (Solarbio) for 60 min at room temperature (RT), tissue sections were incubated in the primary antibody overnight at 4 °C and then stained with FITC- or Cy3-conjugated secondary antibodies for 2 h at RT and counterstained with 4’,6-diamidino-2-phenylindole (DAPI) antifade mounting medium. Images were acquired with a Leica SPII confocal fluorescence microscope (Leica, Wetzlar, Germany).

**Transcriptome sequencing and bioinformatics analysis**. Eight-week-old BKS mice were treated as previous description. SGM-miR146a-Exo, SFP, SGM-miR146a-Exo@SFP were prepared and placed on the wound bed as experimental group (n ≥ 3). The wounds were untreated in control groups (n ≥ 3). After mouse euthanasia by cervical dislocation, skin wound tissue from the back of db mice were carefully cleaned with 75 % ethanol and DEPC-treated water. The wound tissue was then dissected and immediately immersed in 2 mL of cold TRI-reagent (Sigma). RNA extracted from 6 control mice and 6 SGM-miR146a-Exo@SFP mice respectively at Day 3 and Day 7 was used to prepare mRNA libraries following the standard Illumina protocol. Total RNA was isolated and purified using TRIzol reagent (Invitrogen, Carlsbad, CA, USA) following the manufacturer's procedure. The RNA amount and purity of each sample was quantified using NanoDrop ND-1000 (NanoDrop, Wilmington, DE, USA). The RNA integrity was assessed by Bioanalyzer 2100 (Agilent, CA, USA) with RIN number >7.0, and confirmed by electrophoresis with denaturing agarose gel. Poly (A) RNA is purified from 1μg total RNA using Dynabeads Oligo (dT) 25-61005 (Thermo Fisher, CA, USA) using two rounds of purification. Then the poly(A) RNA was fragmented into small pieces using Magnesium RNA Fragmentation Module (NEB, cat.e6150, USA) under 94 °C 5-7min. Then the cleaved RNA fragments were reverse-transcribed to create the cDNA by SuperScript™ II Reverse Transcriptase (Invitrogen, cat. 1896649, USA), which were next used to synthesise U-labeled second-stranded DNAs with E. coli DNA polymerase I (NEB, cat.m0209, USA), RNase H (NEB, cat.m0297, USA) and dUTP Solution (Thermo Fisher, cat. R0133, USA). An A-base is then added to the blunt ends of each strand, preparing them for ligation to the indexed adapters. Each adapter contains a T-base overhang for ligating the adapter to the A-tailed fragmented DNA. Single- or dual-index adapters are ligated to the fragments, and size selection was performed with AMPureXP beads. After the heat-labile UDG enzyme (NEB, cat.m0280, USA) treatment of the U-labeled second-stranded DNAs, the ligated products are amplified with PCR by the following conditions: initial denaturation at 95 °C for 3 min; 8 cycles of denaturation at 98 °C for 15 sec, annealing at 60 °C for 15 sec, and extension at 72 °C for 30 sec; and then final extension at 72 °C for 5 min. The average insert size for the final cDNA library was 300±50 bp. At last, we performed the 2×150 bp paired-end sequencing (PE150) on an Illumina Novaseq™ 6000 (LC-Bio Technology CO., Ltd., Hangzhou, China) following the vendor's recommended protocol. Cutadapt software (https://cutadapt.readthedocs.io/en/stable/, version: cutadapt-1.9) was used to remove the reads that contained adaptor contamination. And after removed the low-quality bases and undetermined bases, we used HISAT2 software (<https://daehwankimlab.github.io/hisat2/>, version: hisat2-2.0.4) to map reads to the genome. The mapped reads of each sample were assembled using StringTie (http://ccb.jhu.edu/software/stringtie/, version: stringtie-1.3.4d. Linux_x86_64) with default parameters. Then, all transcriptomes from all samples were merged to reconstruct a comprehensive transcriptome using gffcompare software (http://ccb.jhu.edu/software/stringtie/gffcompare.shtml,version:gffcompare-0.9.8.Linux_x86_64). After the final transcriptome was generated, StringTie and ballgown (http://www.bioconductor.org/packages/release/bioc/html/ballgown.html) were used to estimate the expression levels of all transcripts and perform expression level for mRNAs by calculating FPKM. The differentially expressed mRNAs were selected with fold change > 2 or fold change < 0.5 and p value < 0.05 by R package edgeR or DESeq2, and then analysis GO enrichment and KEGG enrichment to the differentially expressed mRNAs.

**Statistical analysis.** All results presented are from at least three independent experiments for each condition. Data are expressed as mean ± standard deviation (S.D.). One-way or two-way ANOVA was used to determine the level of significance using GraphPad Prism 9.2 software. Differences were considered statistically significant at *p* < 0.05.

**References**

1. Li, Q.K. et al. Regenerative and reparative effects of human chorion-derived stem cell conditioned medium on photo-aged epidermal cells. *Cell Cycle* **15**, 1144-1155 (2016).

2. Zhang, K. et al. Regenerative and reparative effects of human chorion-derived stem cell conditioned medium on photo-aged epidermal cells. *ACS Appl. Mater. Interfaces* **10**, 30081 (2018).

3. Nakamura,Y. et al. Enhanced wound healing by topical administration of mesenchymal stem cells transfected with stromal cell-derived factor-1. *Biomaterials* **34***,* 9393 (2013).

Supplementary Figures

**
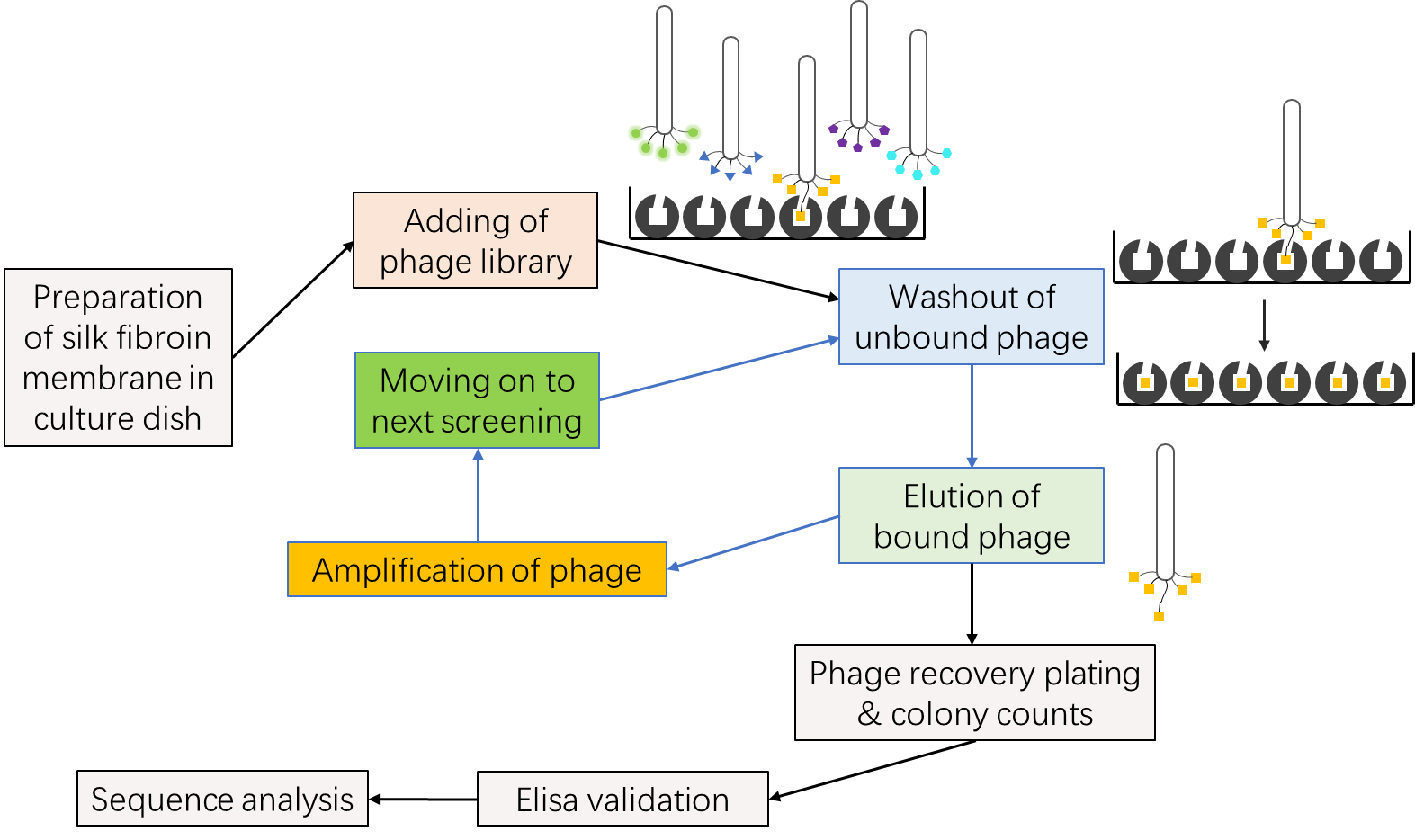
**

**Fig. S1** **Schematic illustration of biopanning of silk fibroin binding protein.**


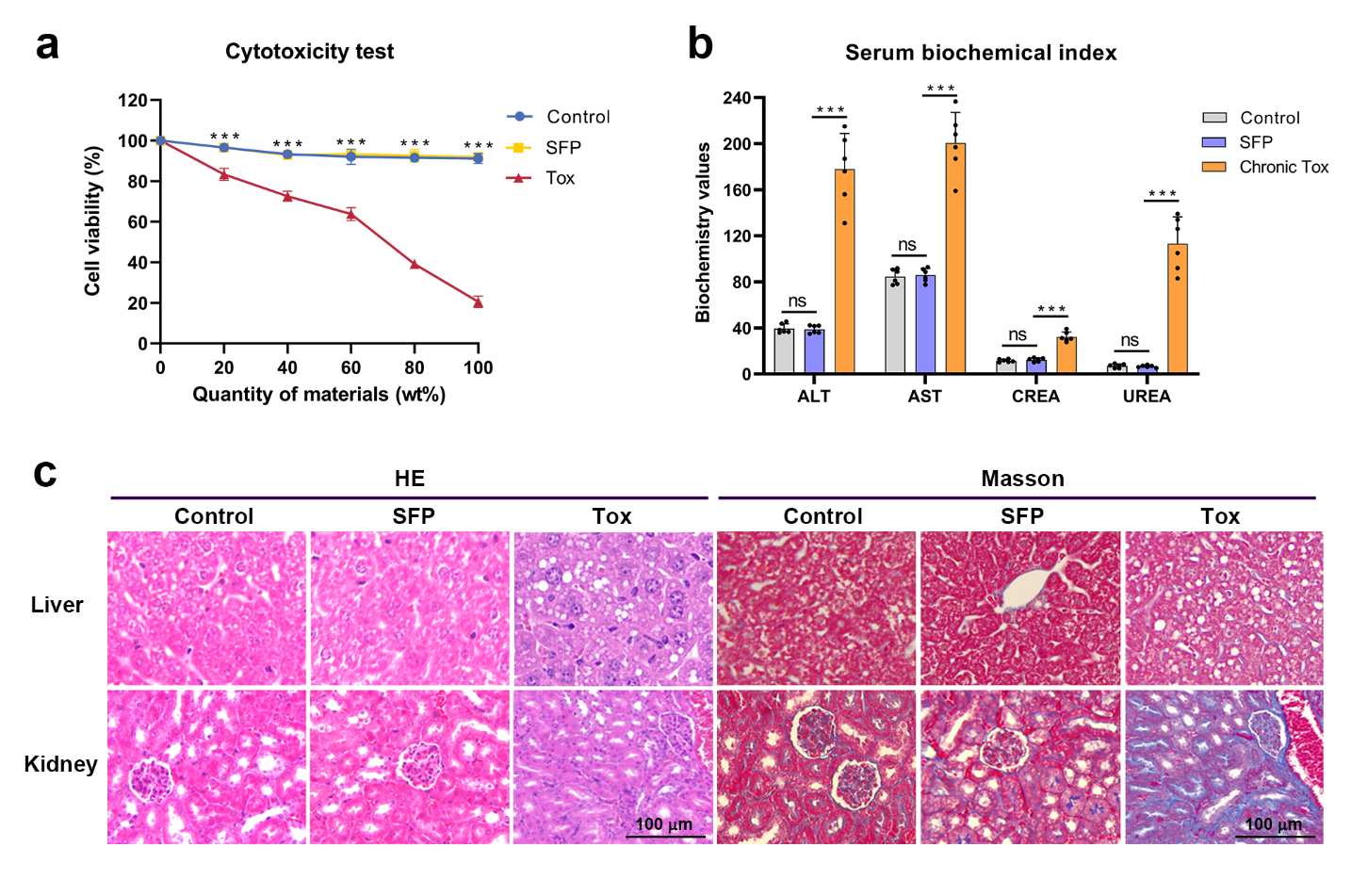


**Fig. S2 The biocompatibility and cytotoxicity tests of the SFP in vitro and in vivo.** (a) SFP had no influence on cell viability detected by CCK8 test (*n* = 6/group). (b) The blood biochemistry indicating hepatorenal function in SFP group were in the normal reference range (*n* = 6/group). (c) The histological sections of liver tissue in Tox group showed fat necrosis. The kidney tissue in Tox group appeared renal tubule edema, glomerular atrophying and interstitial fibrosis hyperplasia (scale bar: 100 μm). There were no obvious abnormalities in SFP and control groups (*n* = 6/group). Data represent the mean ± S.D. of three different experiments (ns: no significance, ****p* < 0.001).


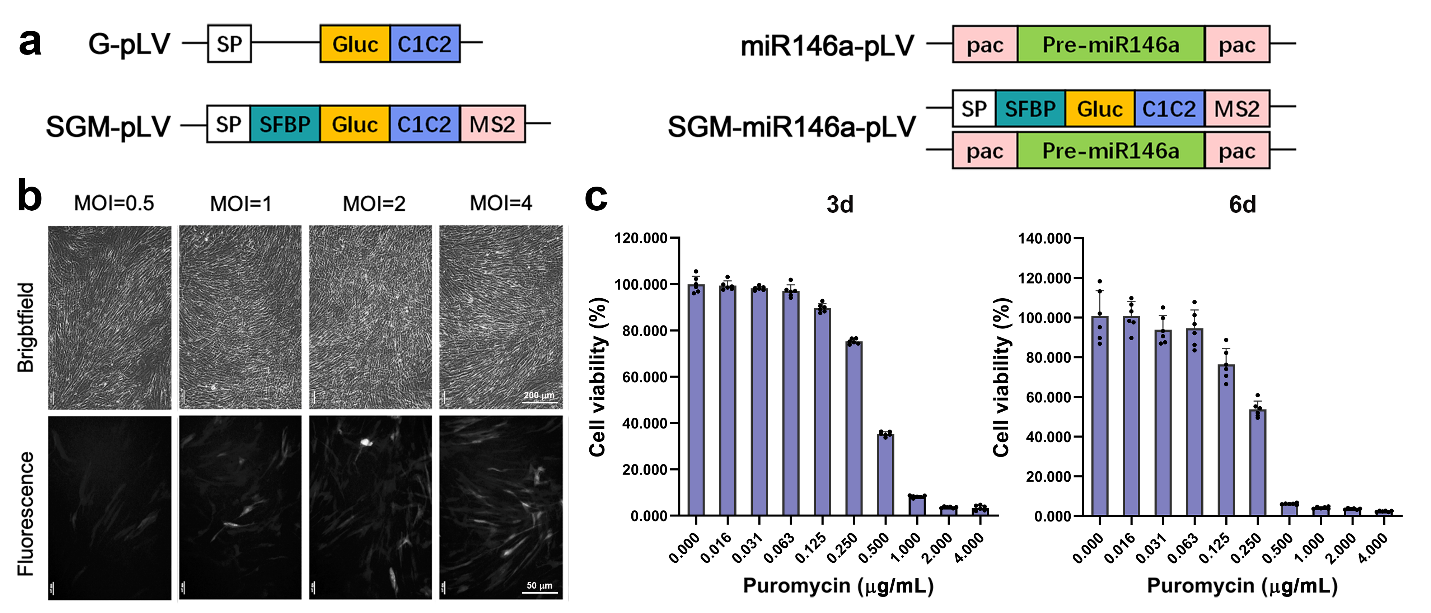


**Fig. S3 Lentiviral transfer plasmids sequence and optimization of lentivirus infection efficiency with resistance screening in PMSCs.** (a) The sequences of recombinant lentiviral transfer plasmids were displayed above. They transfected 293T cells to obtained lentiviral particles. (b) The recombinant lentiviral particles infected PMSCs at the different multiplicity of infection (MOI). (c) Cell viability of PMSCs treated with 0-4 μg/mL purinomycin for 3 days and 6 days. 1 mg/mL was the suitable concentration for puromycin to screen the stable transfected cell line in PMSCs.

**
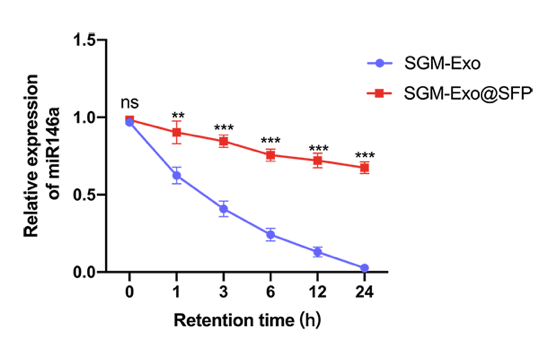
**

**Fig. S4 The protective effects of SGM-Exo@SFP on miR-146a.** The stability of miR-146a in SGM-Exo or SGM-Exo@SFP at 37 °C was assessed by quantitative real-time PCR analysis. The result showed the basic expression of miR-146a could be detected in SGM-Exo and the miR-146a in SGM-Exo@SFP was significantly more stable than that in SGM-Exo. Relative gene expression was normalized to U6 (ns: no significance, ***p* < 0.01, ****p* < 0.001).

**
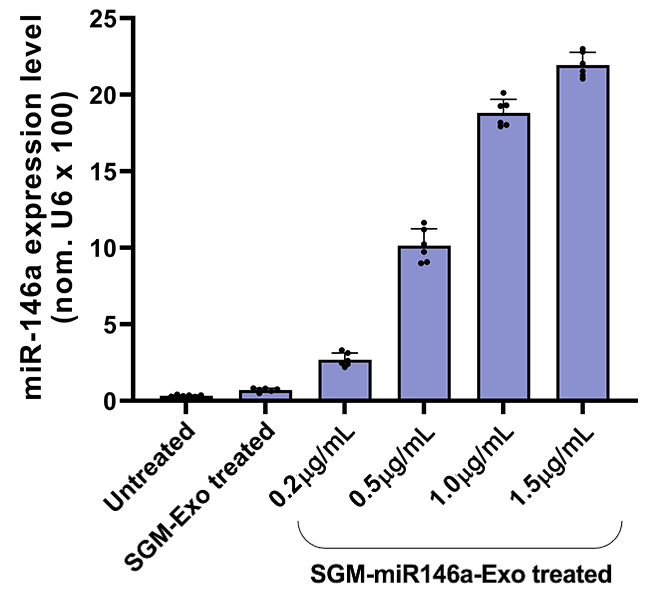
**

**Fig. S5 Dose-dependent relationship between miR-146a expression levels and the amount of SGM-miR146a-Exos used to treat receptor cells.**

**
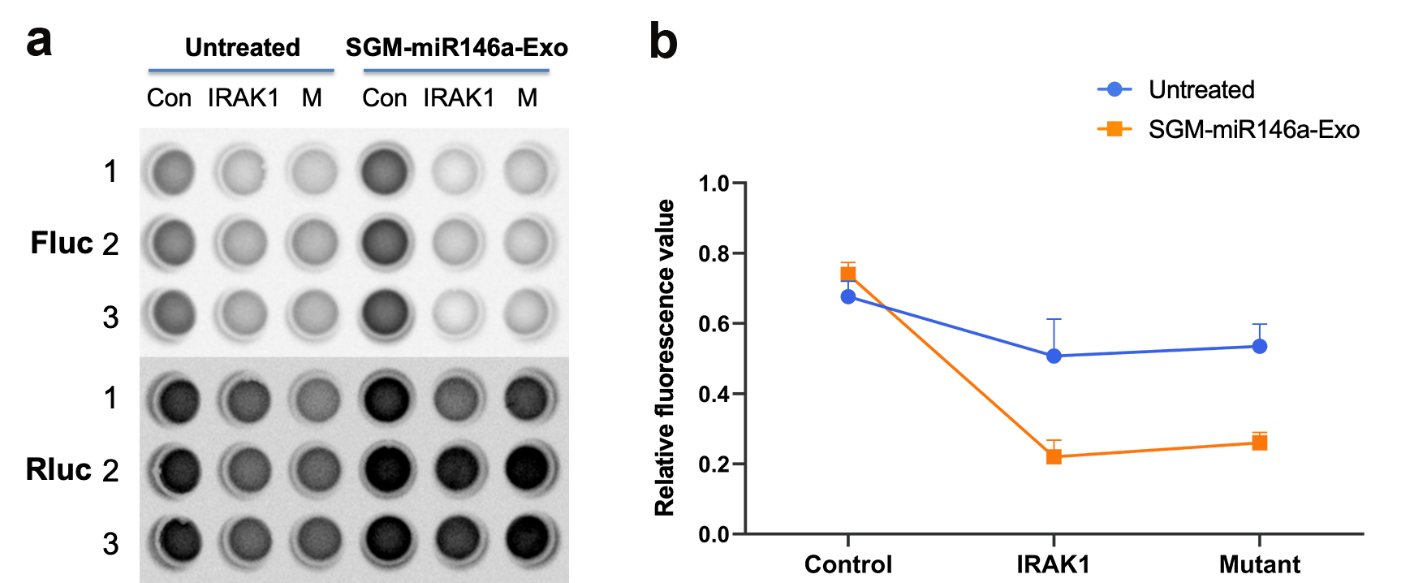
**

**Fig. S6 Suppressive effect of SM-miR146a-Exo on the expression of IRAK1.** The dual-luciferase reporter genes Fluc/Rluc were used to evaluate the inhibitory effect of miR146a on target genes. The expression of Fluc could be inhibited by adding miR146a targeted sequence to its 3 'end. The Rluc as the internal reference. In order to avoid the common substrate effects of Gluc on Fluc and Rluc. Here SGM-Exos and SGM-miR146a-Exos were replaced with SM-Exos and SM-miR146a-Exos without Gluc fusion protein tags. The miR146a target gene was retrieved by Scantarget (www.scantarget.com) to obtain the IRAK1 3'-end target sequence IRAK1-S (S), and further obtain the antisense sequence IRAK1-R (R) and the random mutation sequence Control (Con). (a) The dual-luciferase assay of IRAK1 expression by firefly luciferase (Fluc) and renal luciferase (Rluc). (b) The result showed the fluorescence intensity ratio of Fluc/Rluc was observably reduced in IRAK1-S and IRAK1-R groups. SM-miR146a-Exos could significantly inhibit the expression of miR146a target gene IRAK1.


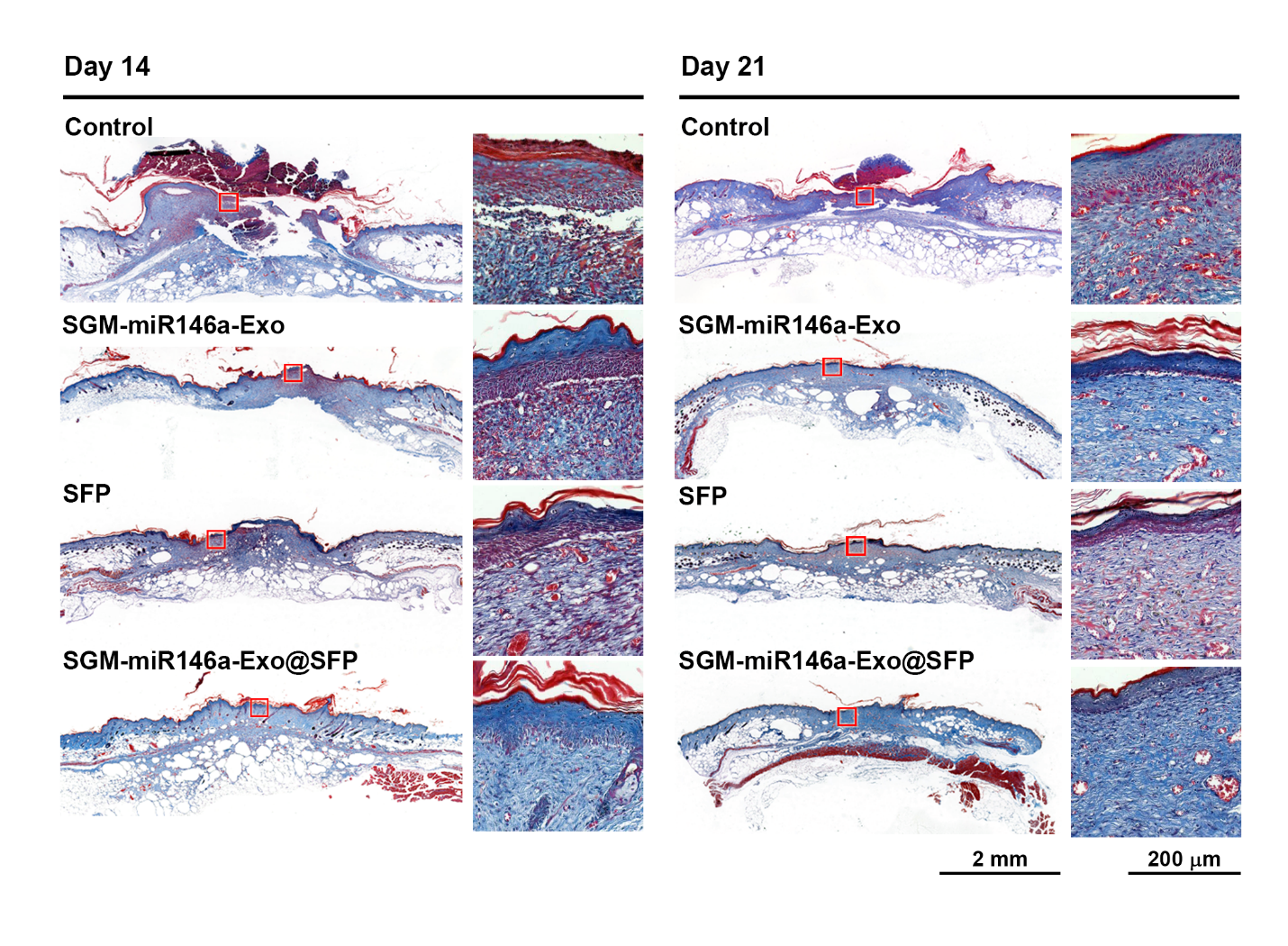


**Fig. S7 SMG-miR146a-Exo@SFP enhanced collagen deposition and remodelling.** Masson's trichrome staining was used to stain collagen fibers and the collagen fiber was stained in blue. At day 14 and day 21 post-injury, the increased collagen deposition was observed in SMG-miR146a-Exo and SMG-miR146a-Exo@SFP groups and the collagen fibers in the SMG-miR146a-Exo@SFP group were normally arranged (left scale bar: 2 mm, right scale bar: 200 μm).


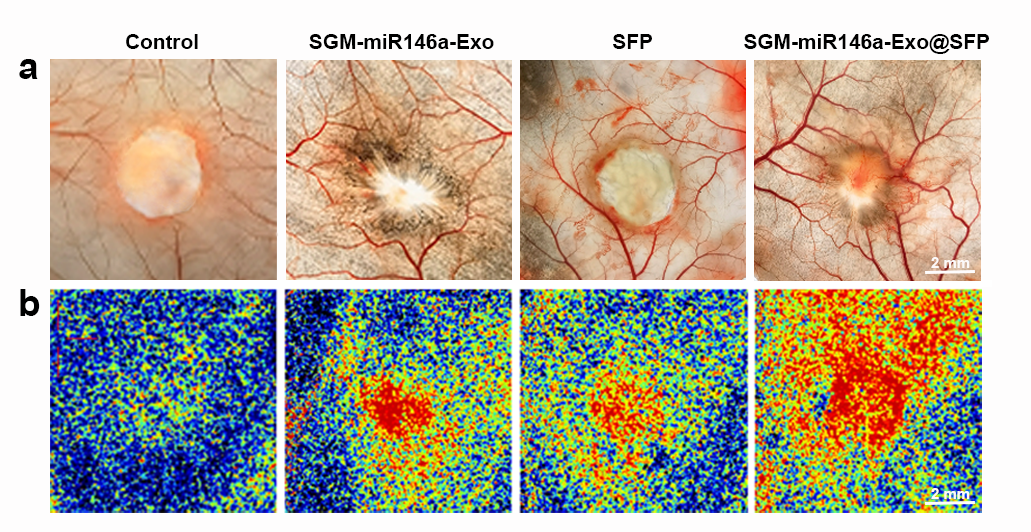


**Fig. S8** **Angiogenesis around the wound at day 14.** (a) The new blood vessels around the wound were notably observed in SGM-miR146a-Exo@SFP group at day 14 by the stereo microscope (scale bar: 2 mm, n = 4/group). (b) The blood flows at the wound sections were evaluated by doppler of animals, flux images of the wounds (scale bar: 2 mm, n = 4/group).


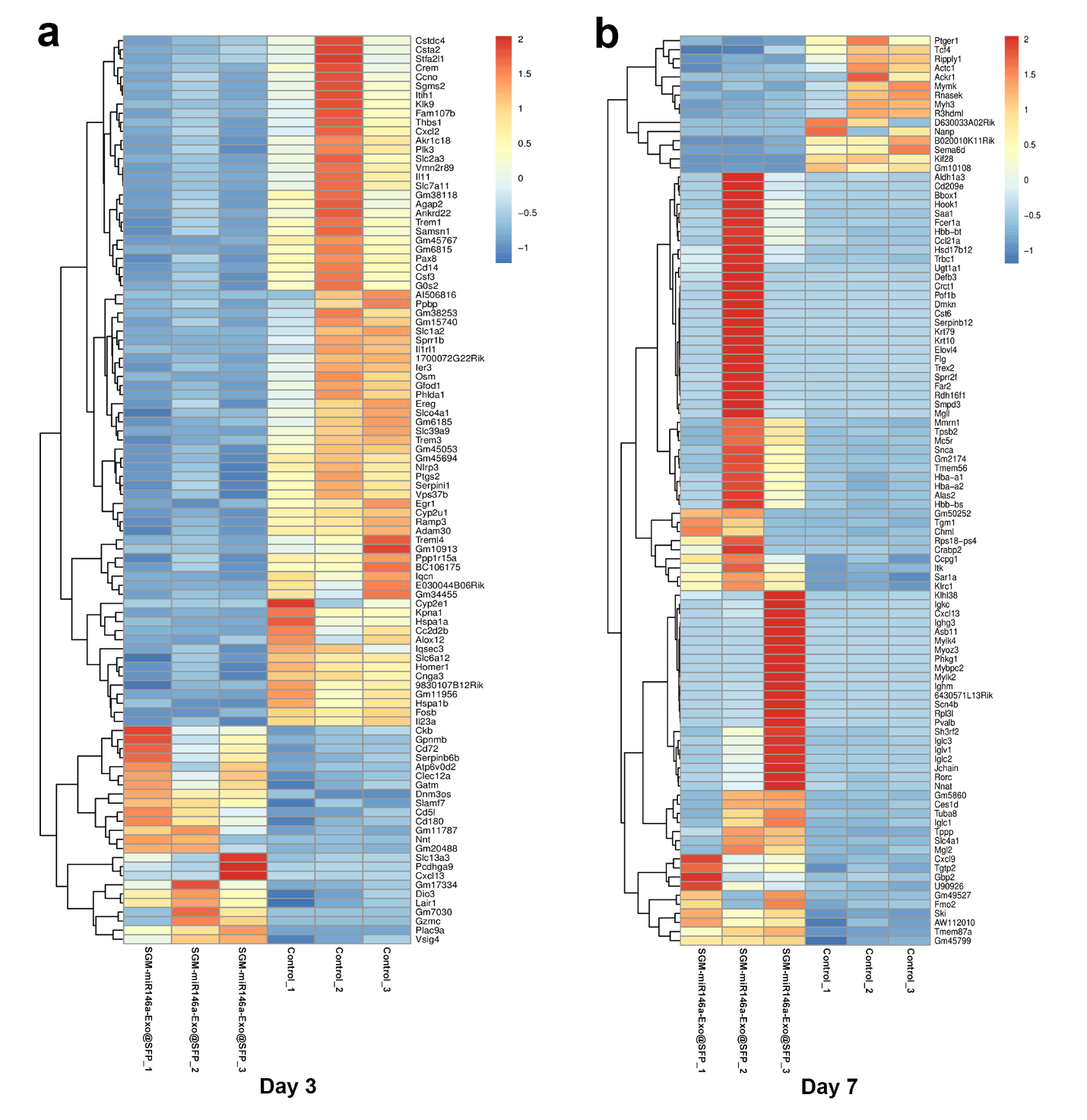


**Fig. S9 Transcriptomics analysis of wounds treated with or without SGM-miR146a-Exo@SFP.** (a) Heatmap of differentially expressed genes (DEGs) at day 3 (SGM-miR146a-Exo@SFP vs. Control). (b) Heatmap of differentially expressed genes (DEGs) at day 7 (SGM-miR146a-Exo@SFP vs. Control).


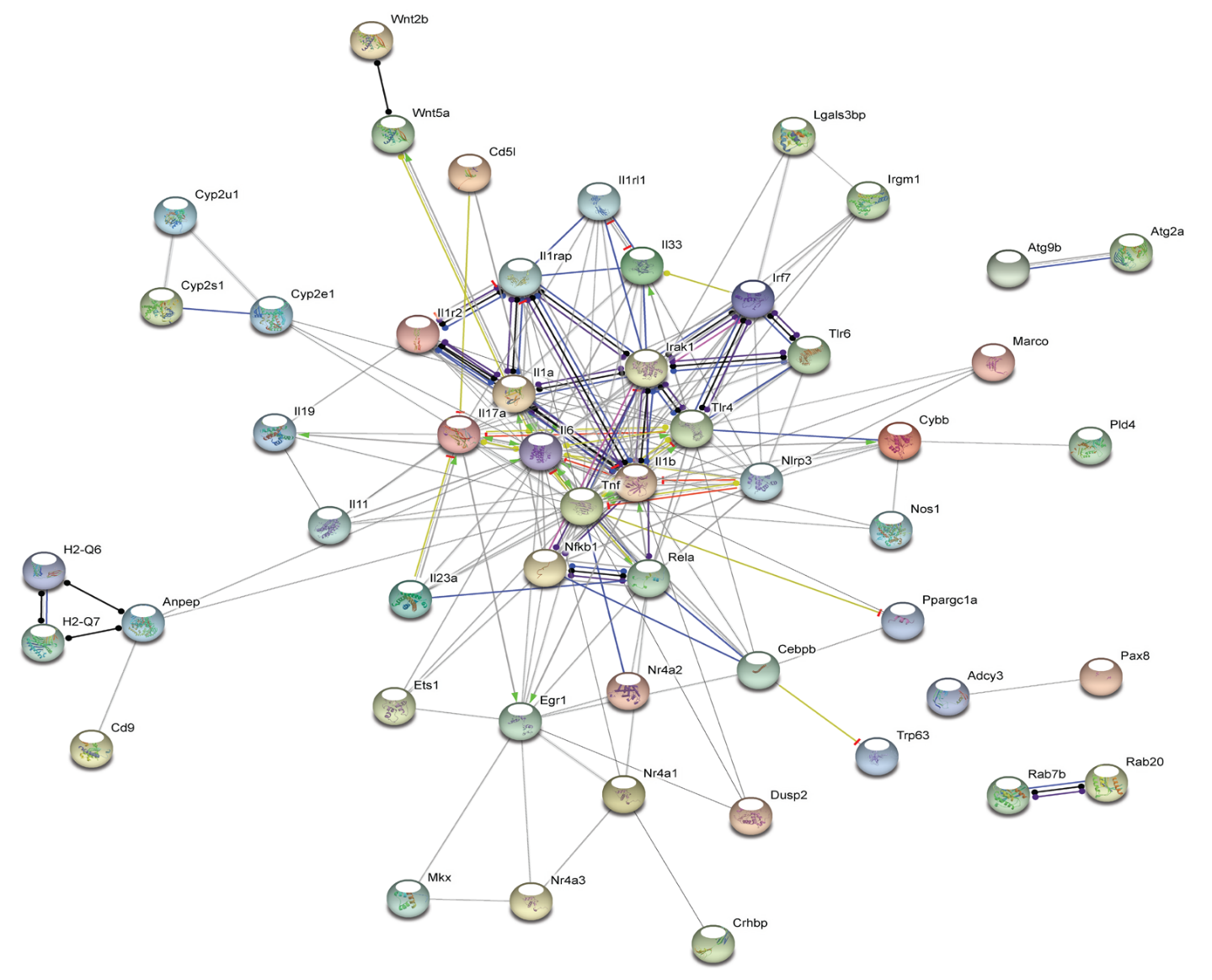


**Fig. S10 Association analysis of 52 DEGs involved in inflammatory reaction and cellular proliferation and differentiation during the wound healing.** The analysis was performed using the OmicStudio tools at https://www.omicstudio.cn/tool.

**
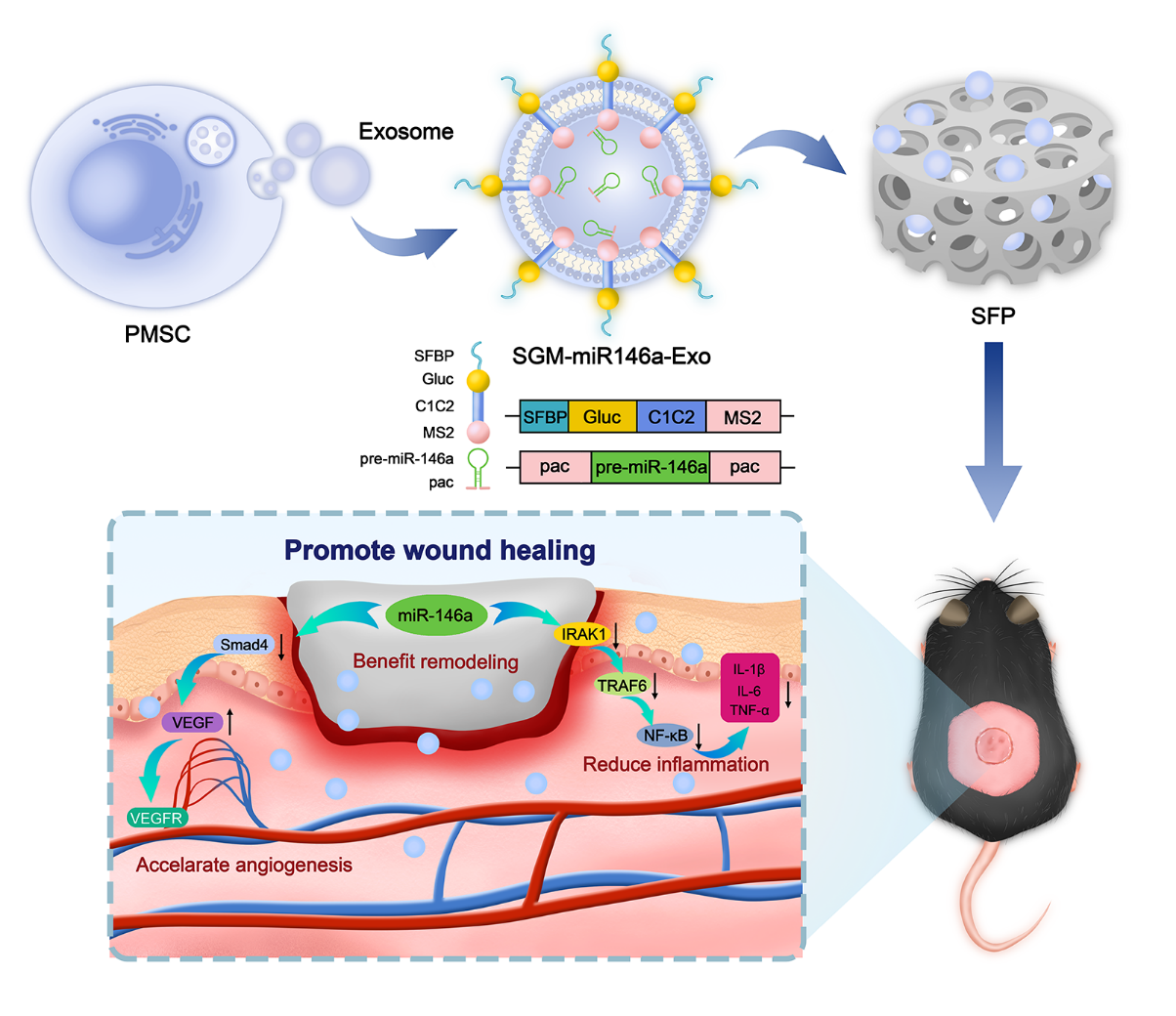
**

**Fig. S11** **Schematic illustration of SGM-miR146a-Exos released from SFP promoting diabetic wound healing.**

Supplementary Tables

**Table S1. Selection conditions for each round in biopanning**

| **Rounds** | **Coated antigen**  **(mg/mL)** | **Washing times** |
| --- | --- | --- |
| 1 | Ag(10) | 5 |
| 2 | Ag (10) | 6 |
| 3 | Ag(10) | 6 |

**Table S2. Results of biopanning**

| **Rounds** | **Input phage (pfu)** | **Output phage (pfu)** |
| --- | --- | --- |
| 1 | 1×10^11^ | 1.5×10^7^ |
| 2 | 1×10^12^ | 1.5×10^8^ |
| 3 | 1×10^12^ | 3.9×10^8^ |

**Table S3. Results of** **polyclonal phage ELISA**

| Phage  (pfu/well) | Round1 | Round2 | Round3 |
| --- | --- | --- | --- |
| 1×10^12^ | 0.765 | 1.090 | 1.323 |
| 3.3×10^11^ | 0.577 | 0.839 | 1.011 |
| 1.1×10^11^ | 0.510 | 0.853 | 0.686 |
| 3.6×10^10^ | 0.501 | 0.515 | 0.603 |
| 1.2×10^10^ | 0.403 | 0.412 | 0.735 |
| 0 | 0.408 | 0.392 | 0.707 |

According to the result, the Round2 output phage was chosen to do the monoclonal screening.

**Table S4. Results of R2P1 output phage ELISA for Ag group**

|  | 1 | 2 | 3 | 4 | 5 | 6 | 7 | 8 | 9 | 10 | 11 | 12 |
| --- | --- | --- | --- | --- | --- | --- | --- | --- | --- | --- | --- | --- |
| A | 0.538 | 0.916 | 0.471 | 0.317 | 0.397 | 0.626 | 1.442 | 0.786 | 0.660 | 1.108 | 0.725 | 0.680 |
| B | 1.255 | 0.394 | 0.473 | 0.453 | 0.449 | 0.474 | 0.862 | 0.717 | 0.697 | 0.723 | 0.717 | 0.591 |
| C | 0.369 | 0.448 | 0.367 | 0.437 | 0.497 | 0.469 | 0.743 | 0.619 | 0.670 | 0.727 | 0.715 | 0.699 |
| D | 0.309 | 0.392 | 0.254 | 0.376 | 0.318 | 0.467 | 0.545 | 0.602 | 0.532 | 0.673 | 0.683 | 0.591 |
| E | 0.309 | 0.356 | 0.380 | 0.464 | 0.372 | 0.491 | 0.617 | 0.589 | 0.583 | 0.774 | 0.754 | 0.686 |
| F | 0.291 | 0.838 | 0.497 | 0.404 | 0.523 | 0.511 | 0.526 | 0.708 | 0.748 | 0.656 | 0.605 | 0.629 |
| G | 0.316 | 0.435 | 0.352 | 0.648 | 0.622 | 1.047 | 0.577 | 1.243 | 0.597 | 1.008 | 0.974 | 0.738 |
| H | 0.505 | 0.448 | 0.417 | 0.789 | 0.687 | 0.491 | 0.801 | 0.729 | 0.694 | 0.674 | 0.784 | 0.767 |

**Table S5. Results of R2P1 output phage ELISA for blank group**

|  | **Blank** |
| --- | --- |
| 1 | 0.474 |
| 2 | 0.551 |
| 3 | 0.424 |
| 4 | 0.469 |
| 5 | 0.566 |
| 6 | 0.510 |
| 7 | 0.495 |
| 8 | 0.505 |
| **average** | 0.499 |

**Table S6. Results of R2P1 output phage ELISA for Ag-blank group**

|  | 1 | 2 | 3 | 4 | 5 | 6 | 7 | 8 | 9 | 10 | 11 | 12 |
| --- | --- | --- | --- | --- | --- | --- | --- | --- | --- | --- | --- | --- |
| A | 0.039 | 0.417 | -0.028 | -0.182 | -0.102 | 0.127 | 0.943 | 0.287 | 0.161 | 0.609 | 0.226 | 0.181 |
| B | 0.756 | -0.105 | -0.026 | -0.046 | -0.05 | -0.025 | 0.363 | 0.218 | 0.198 | 0.224 | 0.218 | 0.092 |
| C | -0.13 | -0.051 | -0.132 | -0.062 | -0.002 | -0.03 | 0.244 | 0.12 | 0.171 | 0.228 | 0.216 | 0.2 |
| D | -0.19 | -0.107 | -0.245 | -0.123 | -0.181 | -0.032 | 0.046 | 0.103 | 0.033 | 0.174 | 0.184 | 0.092 |
| E | -0.19 | -0.143 | -0.119 | -0.035 | -0.127 | -0.008 | 0.118 | 0.09 | 0.084 | 0.275 | 0.255 | 0.187 |
| F | -0.208 | 0.339 | -0.002 | -0.095 | 0.024 | 0.012 | 0.027 | 0.209 | 0.249 | 0.157 | 0.106 | 0.13 |
| G | -0.183 | -0.064 | -0.147 | 0.149 | 0.123 | 0.548 | 0.078 | 0.744 | 0.098 | 0.509 | 0.475 | 0.239 |
| H | 0.006 | -0.051 | -0.082 | 0.29 | 0.188 | -0.008 | 0.302 | 0.23 | 0.195 | 0.175 | 0.285 | 0.268 |

The green clones of the Ag group and blank whose difference was greater than 0.3 for sequencing were selected and to do the secondary verified phage Elisa.

**Table S7. Results of secondary verified phage Elisa for Ag group**

| **clones** | **Parallel group** | **Ag** |
| --- | --- | --- |
| **20000063F-R2P1-A2** | 1 | 0.617 |
|  | 2 | 0.637 |
| **20000063F-R2P1-F2** | 1 | 1.067 |
|  | 2 | 0.982 |
| **20000063F-R2P1-G6** | 1 | 0.925 |
|  | 2 | 0.804 |
| **20000063F-R2P1-A7** | 1 | 1.438 |
|  | 2 | 1.454 |
| **20000063F-R2P1-G8** | 1 | 1.590 |
|  | 2 | 1.385 |
| **20000063F-R2P1-A10** | 1 | 1.191 |
|  | 2 | 1.076 |
| **20000063F-R2P1-G10** | 1 | 0.785 |
|  | 2 | 0.605 |
| **20000063F-R2P1-G11** | 1 | 1.017 |
|  | 2 | 0.925 |

After sequencing, 8 different right sequences were selected and then used in duplicated wells to do the secondary verified phage Elisa.

**Table S8. Results of secondary verified phage Elisa for blank group**

|  | **Blank** |
| --- | --- |
| 1 | 0.517 |
| 2 | 0.587 |
| **average** | 0.55 |

**Table S9. Results of secondary verified phage Elisa for Ag-blank group**

| **clones** |  | **Ag** |
| --- | --- | --- |
| **20000063F-R2P1-A2** | 1 | 0.065 |
|  | 2 | 0.085 |
| **20000063F-R2P1-F2** | 1 | 0.515 |
|  | 2 | 0.430 |
| **20000063F-R2P1-G6** | 1 | 0.373 |
|  | 2 | 0.252 |
| **20000063F-R2P1-A7** | 1 | 0.886 |
|  | 2 | 0.902 |
| **20000063F-R2P1-G8** | 1 | 1.038 |
|  | 2 | 0.833 |
| **20000063F-R2P1-A10** | 1 | 0.639 |
|  | 2 | 0.524 |
| **20000063F-R2P1-G10** | 1 | 0.233 |
|  | 2 | 0.055 |
| **20000063F-R2P1-G11** | 1 | 0.465 |
|  | 2 | 0.373 |

Finally, we got 6 different positive clones from the clones above in Table 9. The 20000063F-R2P1-G8（LSLSPGHFSFVD）was choosen as the SF affinity peptide and its DNA fragment (TTGTCGCTGTCTCCGGGTCATTTTAGTTTTGTGGAT) was designed as silk fibroin binding peptide (SFBP) in the synthetic SFBP-Gluc-C1C2-MS2 (SGM) lentiviral plasmid.

**Table S10. Gluc-lactadherin fusion proteins coding sequence**

| 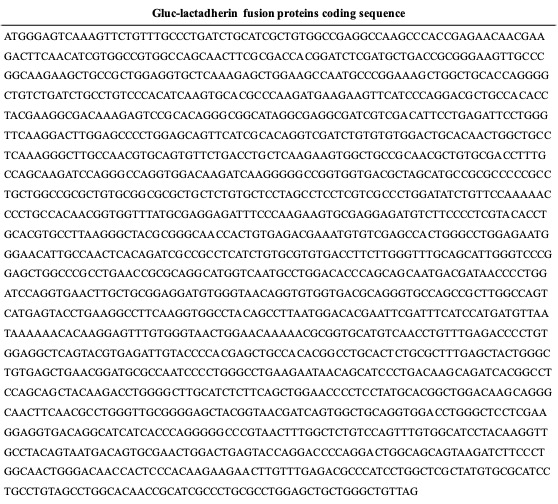 |
| --- |

The Gluc-lactadherin proteins were fused as a bioluminescence report system to indicate exosome activity. Gluc-lactadherin-labeled cell-derived exosomes were used as controls in the detection of exosome activity.

**Table S11. Signal peptide coding sequence**

| 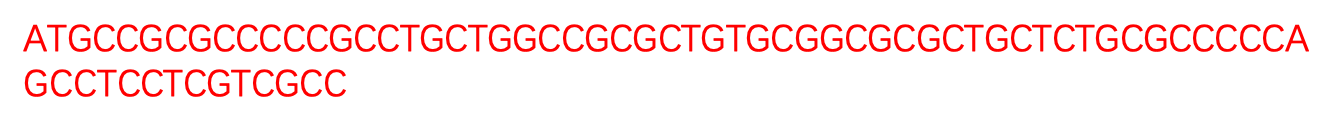 |
| --- |

**Table S12. Silk fibroin binding peptide (SFBP) sequence**

| 3×binding peptide protein sequence |
| --- |
| 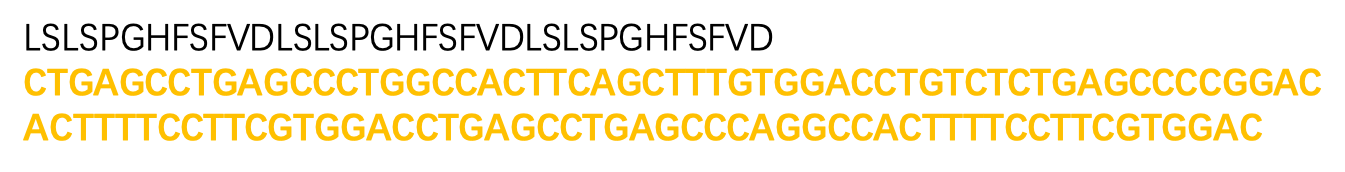 |
| 3×binding peptide coding sequence |
| 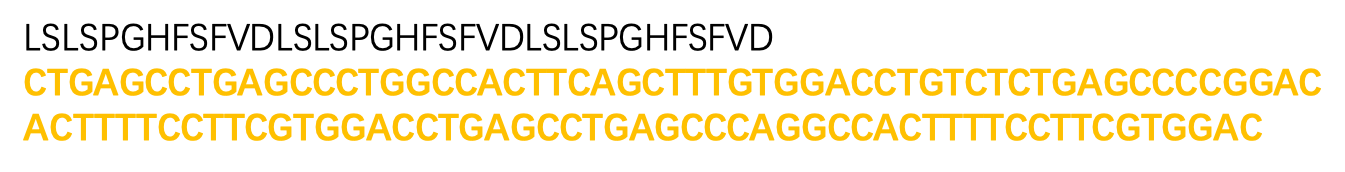 |

**Table S13. C1C2 domain coding sequence**

| 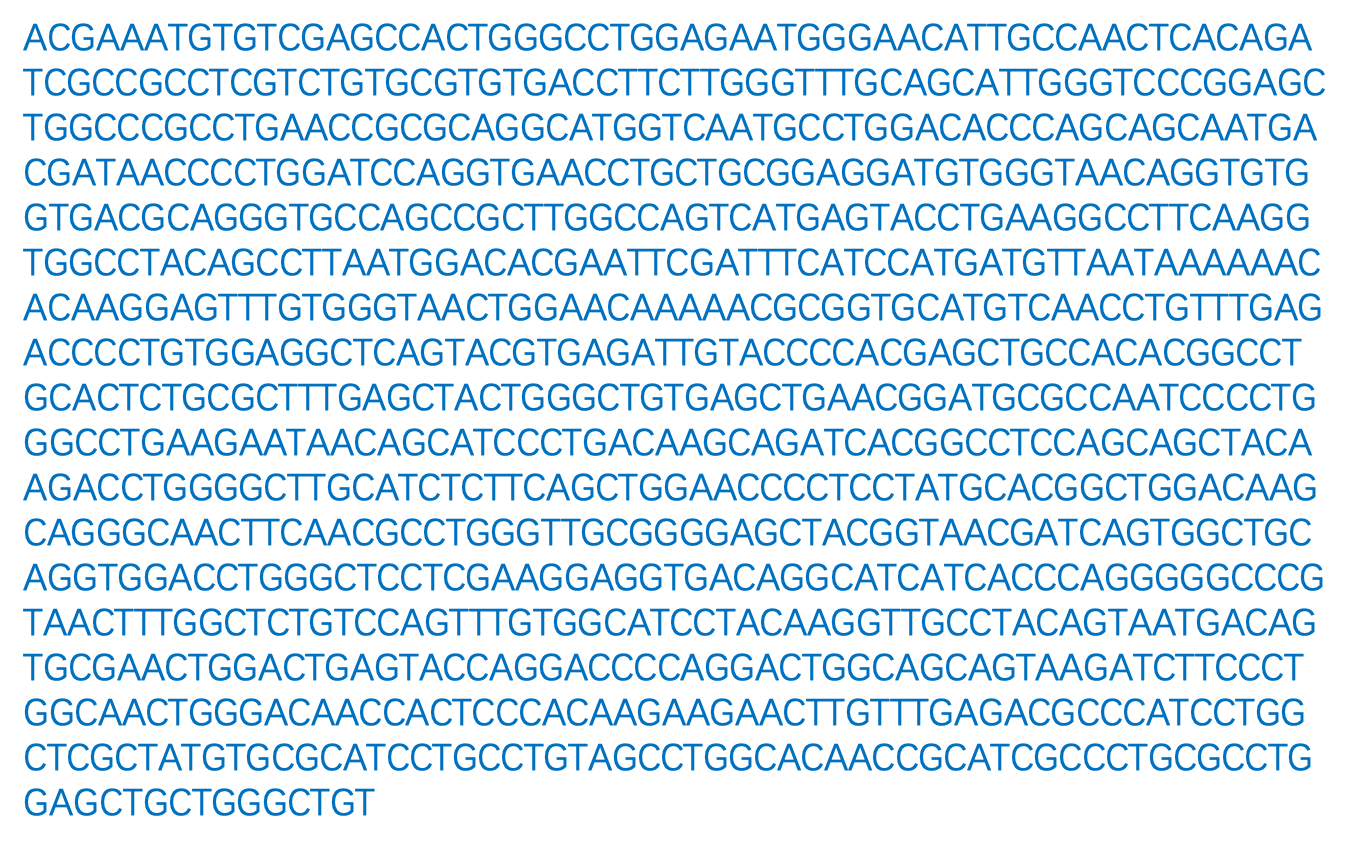 |
| --- |

**Table S14. Gluc domain coding sequence**

| 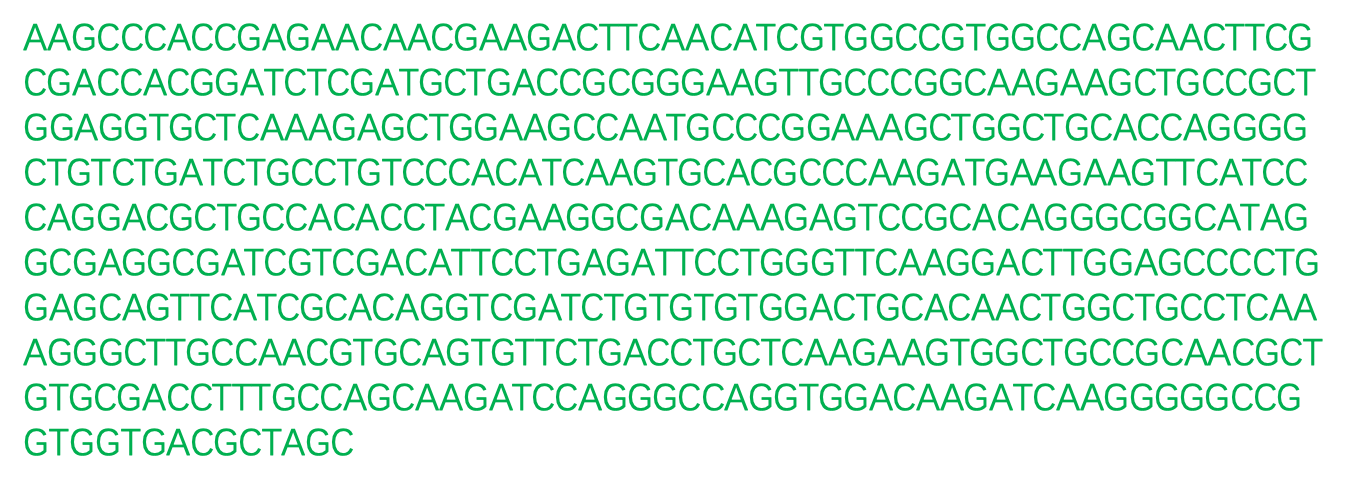 |
| --- |

**Table S15. MS2 coding sequence**

| 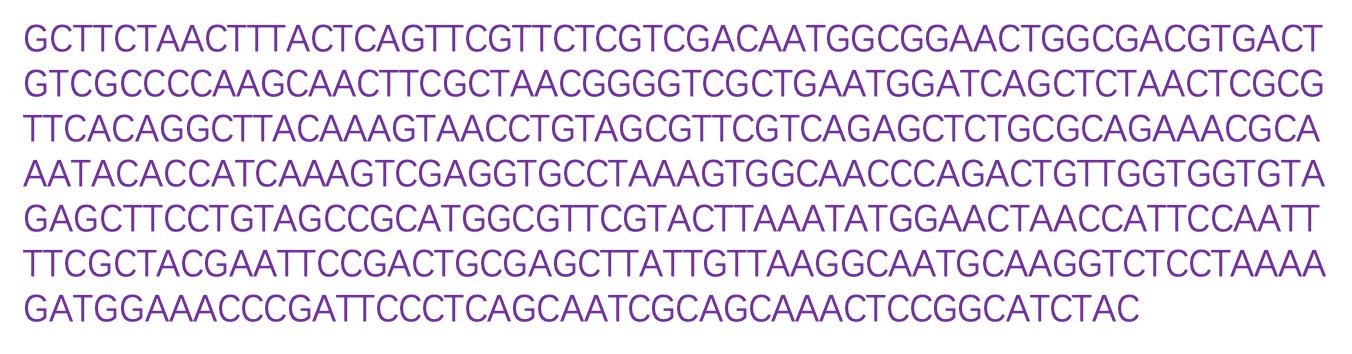 |
| --- |

**Table S16. Construction of the functional fusion protein SGM**

| 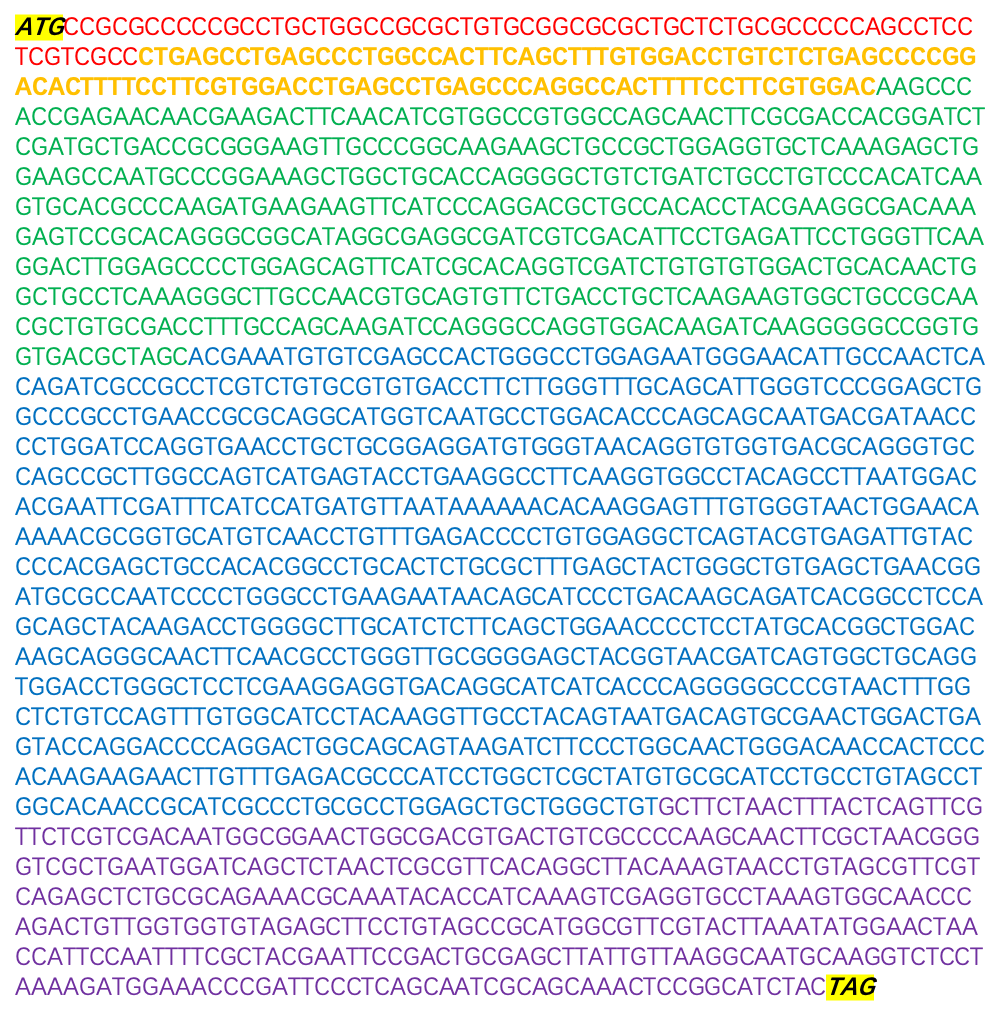 |
| --- |

**Table S17. Abbreviations and functions of engineered exosomes**

| **Exosome** | **Abbreviation** | | **Cell source** | **Function** |  |
| --- | --- | --- | --- | --- | --- |
| exosome | | Exo | PMSC | Control |  |
| Gluc-exosome | | G-Exo | G-PMSC | Gaussia luciferase activity |  |
| SFBP-MS2-exosome | | SM-Exo | SM-PMSC | SF affinity  miRNA-binding |  |
| SFBP-Gluc-MS2-exosome | | SGM-Exo | SGM-PMSC | SF affinity  Gaussia luciferase activity  miRNA-binding |  |
| miR146a-exosome | | miR146a-Exo | miR146a-PMSC | anti-inflammation |  |
| SFBP-Gluc-MS2-miR146a-exosome | | SGM-miR146a-Exo | SGM-miR146a-PMSC | SF affinity  Gaussia luciferase activity  miRNA-binding  anti-inflammation |  |

**Table S18.** **Top 20 DEGs related to inflammation significantly reduced in SGM-miR146a-Exo@SFP group at day 3**

| **Gene** | **Description** | **log2(fc)** | **pval** | **regulation** |
| --- | --- | --- | --- | --- |
| Tlr6 | toll-like receptor 6 [Source:MGI Symbol;Acc:MGI:1341296] | -1.17 | 0.00 | down |
| Tnf | tumor necrosis factor [Source:MGI Symbol;Acc:MGI:104798] | -1.26 | 0.00 | down |
| Tlr4 | toll-like receptor 4 [Source:MGI Symbol;Acc:MGI:96824] | -1.91 | 0.01 | down |
| Il1b | interleukin 1 beta [Source:MGI Symbol;Acc:MGI:96543] | -1.38 | 0.00 | down |
| Il1rl1 | interleukin 1 receptor-like 1 [Source:MGI Symbol;Acc:MGI:98427] | -2.03 | 0.00 | down |
| Il11 | interleukin 11 [Source:MGI Symbol;Acc:MGI:107613] | -2.00 | 0.00 | down |
| Il1r2 | interleukin 1 receptor, type II [Source:MGI Symbol;Acc:MGI:96546] | -1.16 | 0.01 | down |
| Il1a | interleukin 1 alpha [Source:MGI Symbol;Acc:MGI:96542] | -1.22 | 0.03 | down |
| Il19 | interleukin 19 [Source:MGI Symbol;Acc:MGI:1890472] | -2.00 | 0.04 | down |
| Il23a | interleukin 23, alpha subunit p19 [Source:MGI Symbol;Acc:MGI:1932410] | -2.05 | 0.00 | down |
| Fasl | Fas ligand (TNF superfamily, member 6) [Source:MGI Symbol;Acc:MGI:99255] | -2.05 | 0.00 | down |
| Nfkb1 | nuclear factor of kappa light polypeptide gene enhancer in B cells 1, p105 [Source:MGI Symbol;Acc:MGI:97312] | -0.24 | 0.46 | down |
| Cxcr2 | chemokine (C-X-C motif) receptor 2 [Source:MGI Symbol;Acc:MGI:105303] | -1.10 | 0.03 | down |
| Cxcr5 | chemokine (C-X-C motif) receptor 5 [Source:MGI Symbol;Acc:MGI:103567] | -1.69 | 0.00 | down |
| Egr1 | early growth response 1 [Source:MGI Symbol;Acc:MGI:95295] | -1.66 | 0.00 | down |
| Wnt5a | wingless-type MMTV integration site family, member 5A [Source:MGI Symbol;Acc:MGI:98958] | -1.02 | 0.03 | down |
| Osbp2 | oxysterol binding protein 2 [Source:MGI Symbol;Acc:MGI:1921559] | -1.39 | 0.02 | down |
| Atg2a | autophagy related 2A [Source:MGI Symbol;Acc:MGI:1916291] | -1.35 | 0.00 | down |
| Myh2 | myosin, heavy polypeptide 2, skeletal muscle, adult [Source:MGI Symbol;Acc:MGI:1339710] | -4.26 | 0.01 | down |
| Igfn1 | immunoglobulin-like and fibronectin type III domain containing 1 [Source:MGI Symbol;Acc:MGI:3045352] | -3.89 | 0.03 | down |

**Table S19. Top 20 DEGs related to development and differentiation of epidermis significantly increased in SGM-miR146a-Exo@SFP group at day 7**

| **Gene** | **Description** | **log2(fc)** | **pval** | **regulation** |
| --- | --- | --- | --- | --- |
| Cnfn | cornifelin [Source:MGI Symbol;Acc:MGI:1919633] | 3.78 | 0.01 | up |
| Epgn | epithelial mitogen [Source:MGI Symbol;Acc:MGI:1919170] | 2.45 | 0.04 | up |
| Ereg | epiregulin [Source:MGI Symbol;Acc:MGI:107508] | 1.47 | 0.02 | up |
| Epcam | epithelial cell adhesion molecule [Source:MGI Symbol;Acc:MGI:106653] | 3.51 | 0.03 | up |
| Krt10 | keratin 10 [Source:MGI Symbol;Acc:MGI:96685] | 5.51 | 0.00 | up |
| Krt6a | keratin 6A [Source:MGI Symbol;Acc:MGI:1100845] | 3.12 | 0.03 | up |
| Krt6b | keratin 6B [Source:MGI Symbol;Acc:MGI:1333768] | 4.07 | 0.01 | up |
| Sprr1a | small proline-rich protein 1A [Source:MGI Symbol;Acc:MGI:106660] | 4.70 | 0.00 | up |
| Sprr1b | small proline-rich protein 1B [Source:MGI Symbol;Acc:MGI:106659] | 3.04 | 0.02 | up |
| Sprr2a1 | small proline-rich protein 2A1 [Source:MGI Symbol;Acc:MGI:1330350] | 4.84 | 0.00 | up |
| Sprr2a2 | small proline-rich protein 2A2 [Source:MGI Symbol;Acc:MGI:3845026] | 5.23 | 0.01 | up |
| Sprr2b | small proline-rich protein 2B [Source:MGI Symbol;Acc:MGI:1330352] | 4.73 | 0.01 | up |
| Sprr2d | small proline-rich protein 2D [Source:MGI Symbol;Acc:MGI:1330347] | 4.35 | 0.00 | up |
| Sprr2e | small proline-rich protein 2E [Source:MGI Symbol;Acc:MGI:1330346] | 4.18 | 0.00 | up |
| Sprr2f | small proline-rich protein 2F [Source:MGI Symbol;Acc:MGI:1330349] | 5.70 | 0.00 | up |
| Sprr2g | small proline-rich protein 2G [Source:MGI Symbol;Acc:MGI:1330348] | 5.04 | 0.00 | up |
| Sprr2h | small proline-rich protein 2H [Source:MGI Symbol;Acc:MGI:1330343] | 3.11 | 0.03 | up |
| Sprr2i | small proline-rich protein 2I [Source:MGI Symbol;Acc:MGI:1330309] | 4.71 | 0.00 | up |
| Sprr2k | small proline-rich protein 2K [Source:MGI Symbol;Acc:MGI:1330344] | 4.36 | 0.01 | up |
| Pou3f1 | POU domain, class 3, transcription factor 1 [Source:MGI Symbol;Acc:MGI:101896] | 2.54 | 0.05 | up |
| Trp63 | transformation related protein 63 [Source:MGI Symbol;Acc:MGI:1330810] | 2.33 | 0.02 | up |
